# Optimal Translational Termination Requires C4 Lysyl Hydroxylation of eRF1

**DOI:** 10.1016/j.molcel.2013.12.028

**Published:** 2014-02-20

**Authors:** Tianshu Feng, Atsushi Yamamoto, Sarah E. Wilkins, Elizaveta Sokolova, Luke A. Yates, Martin Münzel, Pooja Singh, Richard J. Hopkinson, Roman Fischer, Matthew E. Cockman, Jake Shelley, David C. Trudgian, Johannes Schödel, James S.O. McCullagh, Wei Ge, Benedikt M. Kessler, Robert J. Gilbert, Ludmila Y. Frolova, Elena Alkalaeva, Peter J. Ratcliffe, Christopher J. Schofield, Mathew L. Coleman

**Affiliations:** 1 Centre for Cellular and Molecular Physiology, Oxford University, Roosevelt Drive, Oxford OX3 7BN, UK; 2 Chemistry Research Laboratory, Oxford University, 12 Mansfield Road, Oxford OX1 3TA, UK; 3 Engelhardt Institute of Molecular Biology, the Russian Academy of Sciences, 119991 Moscow, Russia; 4 Division of Structural Biology, Wellcome Trust Centre for Human Genetics, University of Oxford, Oxford OX3 7BN, UK; 5 Target Discovery Institute, Nuffield Department of Medicine, Oxford University, Roosevelt Drive, Oxford OX3 7FZ, UK; 6 Biochemistry Department and Proteomics Core, University of Texas Southwestern Medical Center, Dallas, TX 75390-9038, USA; 7 Department of Nephrology and Hypertension, Friedrich-Alexander-University Erlangen-Nuremberg, Erlangen 91054, Germany

## Abstract

Efficient stop codon recognition and peptidyl-tRNA hydrolysis are essential in order to terminate translational elongation and maintain protein sequence fidelity. Eukaryotic translational termination is mediated by a release factor complex that includes eukaryotic release factor 1 (eRF1) and eRF3. The N terminus of eRF1 contains highly conserved sequence motifs that couple stop codon recognition at the ribosomal A site to peptidyl-tRNA hydrolysis. We reveal that Jumonji domain-containing 4 (Jmjd4), a 2-oxoglutarate- and Fe(II)-dependent oxygenase, catalyzes carbon 4 (C4) lysyl hydroxylation of eRF1. This posttranslational modification takes place at an invariant lysine within the eRF1 NIKS motif and is required for optimal translational termination efficiency. These findings further highlight the role of 2-oxoglutarate/Fe(II) oxygenases in fundamental cellular processes and provide additional evidence that ensuring fidelity of protein translation is a major role of hydroxylation.

## Highlights

• Jmjd4 hydroxylates translational termination factor eRF1
• The C4 lysyl hydroxylase activity of Jmjd4 is unprecedented in animals
• Hydroxylation occurs within the eRF1 stop codon recognition domain
• Inhibiting eRF1 K63 hydroxylation promotes stop codon readthrough

## Graphical Abstract

**Figure:**
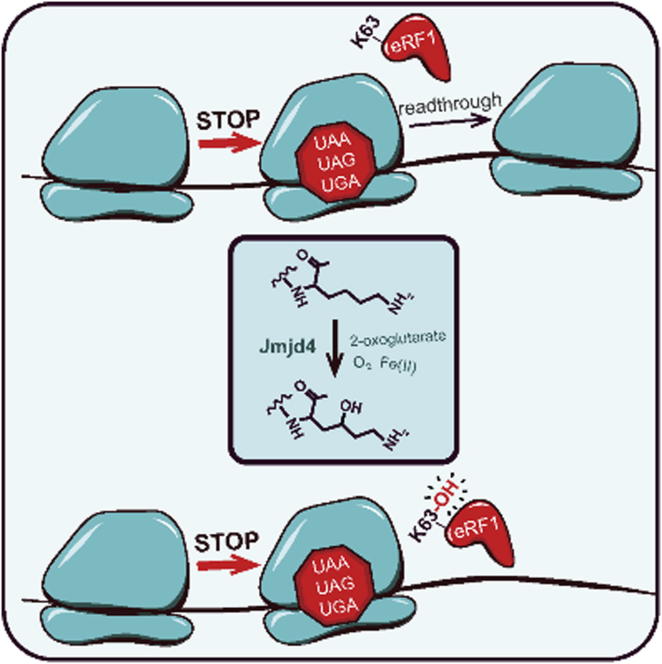

## Teaser

Accurate termination of protein synthesis at a stop codon is essential in order to maintain protein sequence fidelity. Feng et al. show that translational termination is optimized by lysyl hydroxylation of the eukaryotic release factor eRF1 by Jmjd4, a 2-oxoglutarate- and Fe(II)-dependent oxygenase.

## Introduction

Hydroxylation was historically considered a rare posttranslational modification largely restricted to proteins involved in extracellular matrix formation (Walsh, 2005). The discovery of protein hydroxylases that regulate hypoxia signaling (Kaelin and Ratcliffe, 2008), together with widespread ankyrin repeat hydroxylation (Coleman and Ratcliffe, 2009), suggested that this posttranslational modification may in fact be common but relatively poorly characterized.

Many protein hydroxylases belong to the family of 2-oxoglutarate (2OG)- and Fe(II)-dependent oxygenases (2OG oxygenases) that oxidizes diverse substrates, including lipid, nucleic acid, and small molecules (Kaelin and Ratcliffe, 2008; Klose et al., 2006; Loenarz and Schofield, 2011). Since these enzymes also require molecular oxygen for activity, they have the potential to act as oxygen sensors. A family of prolyl hydroxylases (PHD1–PHD3) that targets hypoxia-inducible transcription factor (HIF-α) for proteasomal degradation is inactivated by hypoxia (Kaelin and Ratcliffe, 2008; Loenarz and Schofield, 2008). In addition to their role in hypoxia signaling, 2OG oxygenases are involved in a variety of other fundamental cellular processes, such as chromatin remodeling. A subfamily of 2OG oxygenases with a common JmjC catalytic domain catalyzes histone demethylation via a hydroxylation reaction (Klose et al., 2006; Loenarz and Schofield, 2008).

The role of 2OG oxygenases in regulating HIF-α transcriptional activity and histone demethylation is consistent with the emerging role for these enzymes in regulating gene expression. Jmjd6 is a JmjC 2OG oxygenase that catalyzes carbon 5 (C5) lysyl hydroxylation of arginine/serine-rich (RS) domain splicing factors and histones (Unoki et al., 2013; Webby et al., 2009). Recent reports suggest that 2OG oxygenases may also regulate gene expression at the level of protein synthesis. AlkbH8 hydroxylates 5-methoxycarbonylmethyluridine at the wobble position of tRNA anticodons (Fu et al., 2010; van den Born et al., 2011). Similarly, TYW5 catalyzes the hydroxylation of a hypermodified guanosine in the phenylalanine tRNA anticodon (Noma et al., 2010). Furthermore, two related JmjC 2OG oxygenases, MINA53 and NO66, hydroxylate ribosomal proteins Rpl27a and Rpl8, respectively, at specific histidyl residues proximal to the peptidyl transferase center (Ge et al., 2012). Whether other 2OG oxygenases also regulate protein synthesis is unknown but would be of considerable interest considering the importance of protein translation in gene expression regulation and disease.

In this study, we identify an uncharacterized JmjC 2OG oxygenase, Jmjd4, as a regulator of translational termination—a fundamental cellular process required for decoding stop codons and maintaining protein sequence fidelity. We show that Jmjd4 optimizes translational termination via lysyl hydroxylation of the stop codon recognition domain of an essential release factor termed eRF1. Jmjd4 regulates translational termination via hydroxylation of K63 at the C4 position of the side chain. C4 lysyl hydroxylation represents a posttranslational modification not previously widely described in animals.

## Results

### Jmjd4 Interacts with Translational Termination Factor eRF1

**Figure 1:**
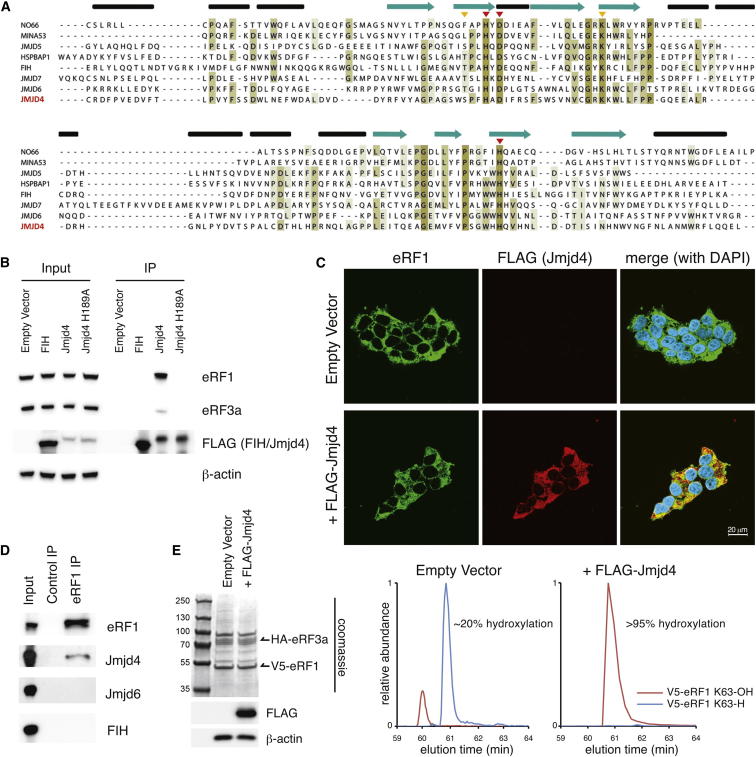
Jmjd4 Interacts with the eRF1/eRF3a Translational Termination Complex in an Activity-Dependent Manner (A) Protein sequences of human JmjC domains were aligned and shaded using Jalview. The Jmjd6 secondary structure, as defined by crystallographic analysis (Mantri et al., 2010), is indicated with α helices (cylinders) and β strands (arrows). The conserved double-stranded β helix core is in cyan (arrows). Triangles indicate residues binding Fe(II) (red) and 2OG (yellow). Mutation of the first Fe(II)-binding residue (His189 in Jmjd4) is predicted to inhibit activity. (B) Anti-FLAG immunoprecipitates of cell extracts from the indicated HEK293T cell lines were immunoblotted for endogenous eRF1 and eRF3a. Input (5%) = cell extract prior to immunoprecipitation (IP). eRF1 levels were quantified by densitometry analysis using NIH ImageJ. (C) HEK293T cell lines were immunostained for eRF1 (green) and FLAG-Jmjd4 (red). Nuclei were visualized with DAPI (blue). (D) Endogenous eRF1 and Jmjd4 interact. eRF1 was immunoprecipitated from HEK293T extracts prior to immunoblot for the indicated proteins. (E) Overexpressed Jmjd4 promotes hydroxylation of overexpressed eRF1 at K63. Left: Coomassie gel showing 5% input following anti-V5 purification of the V5-eRF1/HA-eRF3a complex from HEK293T cells overexpressing empty vector or FLAG-Jmjd4 (immunoblot bottom panel). The remainder of the sample (95%) was digested with Arg-C in-solution prior to LC-MS analyses. The chromatograms indicate the elution time and relative abundance of extracted ion masses corresponding to unhydroxylated (blue) and K63-hydroxylated (red) eRF1_48–65_ ([M+H]^3+^; K63-H: *m/z* 646.68; K63-OH: *m/z* 652.00) in the absence (middle) and presence (right) of FLAG-Jmjd4. See also Figure S1.

Ribosomal hydroxylases, Jmjd6, and the asparaginyl hydroxylase FIH (factor inhibiting HIF) belong to a subfamily of 2OG oxygenases that share a JmjC catalytic domain related to that in 2OG-dependent histone demethylases (Ge et al., 2012; Klose et al., 2006) (Figure S1A available online). We hypothesized that uncharacterized enzymes of this family would include regulators of gene expression. Jmjd4 is a highly conserved putative 2OG oxygenase that contains residues required for enzymatic activity and shares 34% sequence identity with that of the lysyl hydroxylase Jmjd6 (Figures 1A, S1A, and S1B). To investigate the function of Jmjd4, we screened for substrates by identifying proteins that only interact with the wild-type active enzyme. Affinity purification of FLAG-Jmjd4 and mass spectrometry (MS) did not identify known Jmjd6 substrates (Unoki et al., 2013; Webby et al., 2009), RS domain proteins, or histones (data not shown). The most abundant activity-dependent Jmjd4 interactors were eRF1 and eRF3a (Figure S1C), which are nonredundant proteins required for stop codon recognition and translational termination (Kisselev and Buckingham, 2000; Nakamura and Ito, 2011). Immunoblot analysis confirmed that the eRF1/eRF3a complex specifically interacts with wild-type FLAG-Jmjd4 (Figure 1B), but not a Jmjd4 mutant predicted to be inactive due to defective Fe(II) binding (H189A; see Figure 1A) or FIH. The stoichiometry of binding indicated that eRF1, rather than eRF3a, likely interacts directly with FLAG-Jmjd4 (Figures 1B and S1C). Consistent with their interaction, FLAG-Jmjd4 and endogenous eRF1 are cytoplasmically localized in human embryonic kidney 293T (HEK293T) cells (Figure 1C). Furthermore, endogenous eRF1 specifically interacts with endogenous Jmjd4, but not FIH or Jmjd6 (Figure 1D).

### Jmjd4 Is a 2-Oxoglutarate/Fe(II)-Dependent Oxygenase that Hydroxylates eRF1 at K63

**Figure 2:**
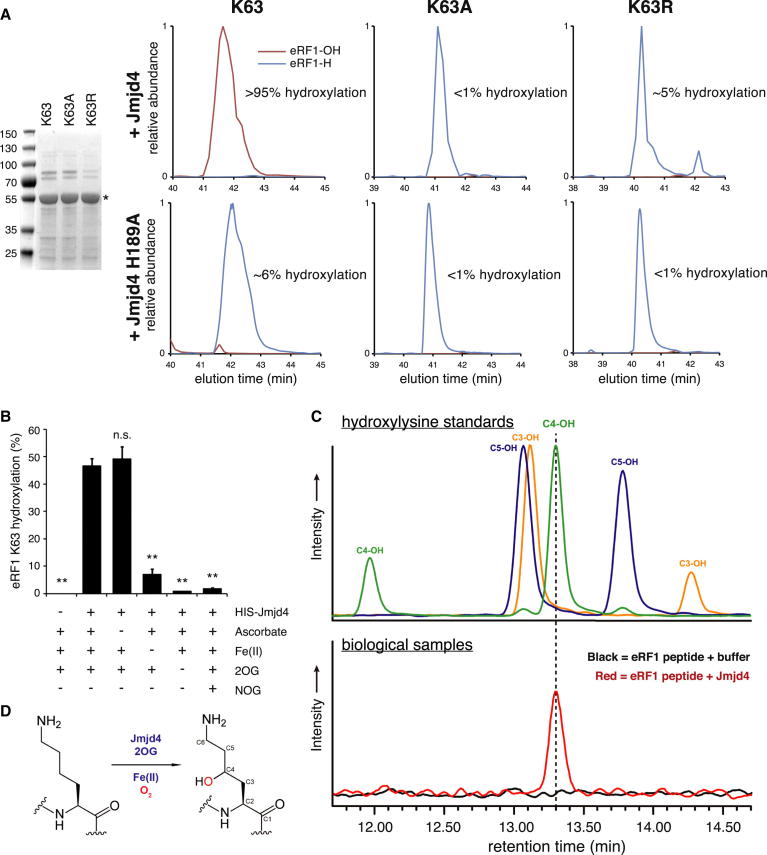
Jmjd4 Catalyzes 2OG- and Fe(II)-Dependent C4 Lysyl Hydroxylation of eRF1 (A) K63 mutation prevents Jmjd4-dependent hydroxylation of eRF1. Left: Coomassie gel of partially purified recombinant eRF1 and mutants. Right: LC-MS extracted ion chromatograms (EICs) show wild-type and mutant eRF1 reacted with either wild-type (top row) or mutant H189A (bottom row) Jmjd4. ([M+H]^2+^; K63-H: *m/z* 698.842; K63-OH: *m/z* 706.840; K63R-H: *m/z* 712.846; K63R-OH: *m/z* 720.843; K63A-H: *m/z* 791.880; K63A-OH: *m/z* 799.878). (B) Jmjd4 is a 2OG/Fe(II)-dependent oxygenase. In vitro assays were performed in the presence or absence of the indicated cofactors and inhibitors. 2OG oxygenases are competitively inhibited by NOG, a nonhydrolysable form of 2OG. Data represent mean ± SEM. Statistical significance was evaluated by ANOVA followed by Dunett’s post hoc test, comparing all treatments to the reaction complemented with all cofactors (n.s., not significant; ^∗∗^p < 0.01). (C) Jmjd4 is a C4 lysyl hydroxylase. Bottom: a cyclic thioether-linked dimer of a 15-mer peptide containing eRF1 residues 57–70 was untreated (buffer, black) or Jmjd4 hydroxylated (red) prior to hydrolysis and LC-MS. Top: chromatography peaks observed in biological samples were identified with C3, C4, and/or C5 hydroxylysine standards. Note that two peaks are observed because each standard is a mixture of stereoisomers. See Figure S2 for NMR of standards and further validation of the C4 assignment. (D) Schematic of C4 lysyl hydroxylation catalyzed by Jmjd4. See also Figure S2.

To test whether Jmjd4 hydroxylates eRF1 and/or eRF3a, overexpression vectors were cotransfected into HEK293T cells: anti-V5 purification of the V5-eRF1/HA-eRF3a complex, followed by proteolysis and MS analysis, identified a single hydroxylation site in eRF1 at K63 (Figure S1D), with 20% hydroxylation in control cells and >95% in cells overexpressing FLAG-Jmjd4 (Figure 1E). These data indicate that exogenous FLAG-Jmjd4 can promote lysyl hydroxylation of overexpressed eRF1 in cells. To test whether Jmjd4 catalyzes eRF1 hydroxylation directly, we incubated recombinant Jmjd4 and eRF1 in the presence of cofactors in vitro; eRF1 K63 hydroxylation was efficiently catalyzed by wild-type Jmjd4, but not the inactive H189A mutant (Figure 2A). In contrast to wild-type eRF1, K63A and K63R mutants were not efficiently hydroxylated (Figure 2A), thus confirming the specificity of Jmjd4 and further assigning the position of eRF1 hydroxylation.

Previously characterized 2OG oxygenases depend on key nutrients and metabolites for activity, including oxygen, Fe(II), 2OG, and in some cases ascorbate (Loenarz and Schofield, 2008). To explore the enzymatic activity of Jmjd4 in more detail, we performed in vitro hydroxylation assays in the presence or absence of known cofactors and inhibitors. Under these conditions eRF1 K63 hydroxylation was significantly impaired by a 2OG competitive inhibitor (NOG, *N*-Oxalylglycine) or the absence of either Fe(II) or 2OG (Figure 2B). These data confirm that Jmjd4 is a bona fide 2OG- and Fe(II)-dependent oxygenase.

### Jmjd4 Is a C4 Lysyl Hydroxylase

Known lysyl hydroxylases (Jmjd6 and collagen hydroxylases) catalyze hydroxylation at C5 of the amino acid side chain (Webby et al., 2009). In order to investigate the target specificity of Jmjd4, we first attempted to hydroxylate a 24-mer eRF1 peptide for amino acid analyses. However, in contrast to full-length recombinant eRF1 (Figures 2A and 2B), quantitative MS provided little evidence for hydroxylation (<5%), even when using stoichiometric Jmjd4 and saturating levels of cofactors. We postulated that this was due to inappropriate conformational presentation of the linear peptide at the Jmjd4 active site. Crystallographic analyses reveal that K63 is at the apex of a tight turn between two antiparallel α helices (Figure S2A) (Song et al., 2000). In an effort to obtain a mimic of the structurally observed conformation, we analyzed the eRF1 crystal structure and proposed that appropriately cyclized eRF1 peptide fragments may be more efficient substrates (Figure S2B). We found that a thioether-linked dimer (containing eRF1 residues 57–70 twice) supported relatively efficient hydroxylation at both K63 sites by Jmjd4, whereas Jmjd6 did not catalyze hydroxylation, demonstrating different specificities of the two hydroxylases (Figure S2C). Comparison of chemically synthesized C3 and C4 hydroxylysine standards (for NMR see Figures S2D and S2E, respectively) to commercially available C5 hydroxylysine and the K63-hydroxylated eRF1 cyclic peptide indicated that in contrast to known 2OG-dependent lysyl hydroxylases, Jmjd4 is in fact a C4 lysyl hydroxylase (Figures 2C, 2D, and S2F). These findings indicate that Jmjd4 is a 2OG oxygenase that catalyzes a posttranslational modification not widely described in animals previously.

### Endogenous eRF1 K63 Hydroxylation Is Abundant, Ubiquitous, and Dependent on Jmjd4 Enzyme Activity

**Figure 3:**
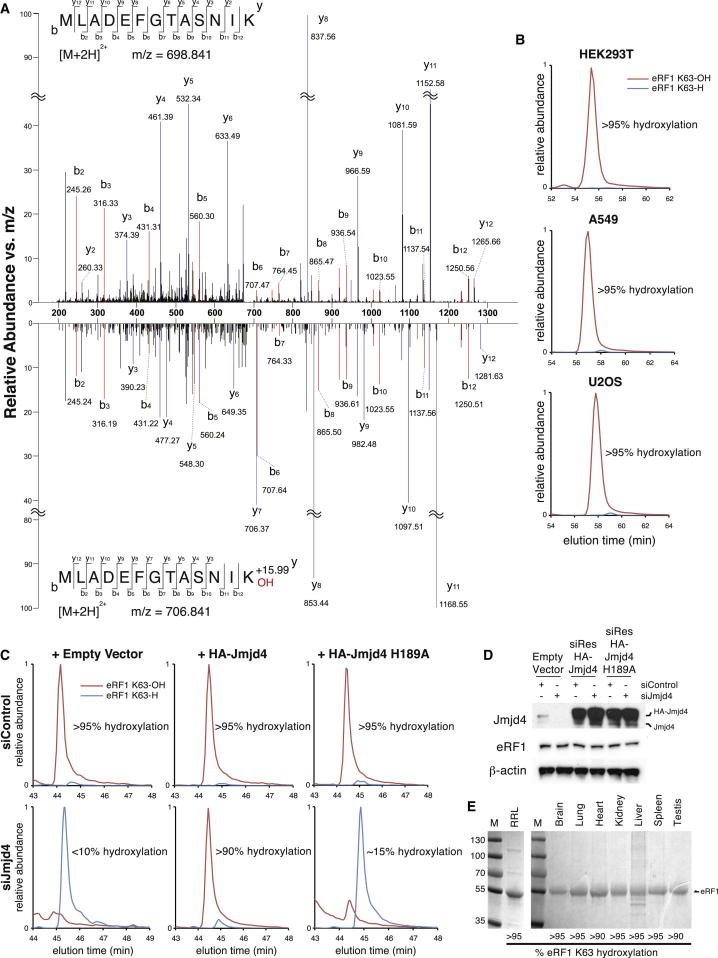
Endogenous eRF1 K63 Hydroxylation Is Abundant, Ubiquitous, and Dependent on Jmjd4 Activity (A) HEK293T eRF1 was trypsinized prior to MS/MS. Spectra show a +16 Da mass shift in all detected y ion fragments (y_2_–y_12_) (blue) when comparing the upper (unhydroxylated) and lower (hydroxylated) panels. In contrast, the masses of detected b ion fragments (b_2_–b_12_) (red) are consistent with their predicted values, indicating K63 hydroxylation. This was confirmed with Arg-C proteolysis (Figure S3A). (B) LC-MS analyses of trypsinized eRF1 indicate abundant K63 hydroxylation in multiple cell types. EICs show relative abundance of unhydroxylated (blue) versus K63 hydroxylated (red) eRF1_51–63_ ([M+H]^2+^; K63-H: *m/z* 698.842; K63-OH: *m/z* 706.840). (C) EICs demonstrate that eRF1 hydroxylation is dependent on Jmjd4 activity ([M+H]^2+^; K63-H: *m/z* 701.852; K63-OH: *m/z* 709.850). The masses are +3 Da relative to (B) due to SILAC (stable isotope labeling by/with amino acids in cell culture) with K^+6^. HeLa cells expressing empty vector (left), siRNA-resistant HA-Jmjd4 (middle), or siRNA-resistant HA-Jmjd4 H189A mRNAs (right) were transfected with control (top row) or Jmjd4 siRNA (bottom row) prior to SILAC labeling and LC-MS quantitation of hydroxylation in newly synthesized eRF1. Note that although hydroxylation of newly synthesized eRF1 is <15% following Jmjd4 siRNA, total eRF1 is ∼50% hydroxylated due to persisting eRF1 synthesized prior to siRNA (data not shown). (D) Cell extracts from (C) were immunoblotted for the indicated proteins. eRF1 levels were quantified by densitometry analysis. (E) Similar analyses in rodent tissues indicate that eRF1 hydroxylation is physiologically relevant and conserved. Rabbit reticulocyte lysate (RRL) and the indicated mouse tissues were diluted or homogenized in lysis buffer prior to eRF1 purification, trypsinolysis, and MS analyses. See also Figure S3.

Next, we aimed to characterize the hydroxylation of endogenous eRF1. Immunopurification of endogenous eRF1 from HEK293T cells followed by tandem mass spectrometry (MS/MS) sequencing of tryptic (Figure 3A) or Arg-C (Figure S3A) protease fragments confirmed that endogenous eRF1 is hydroxylated at K63. Liquid chromatography (LC)-MS quantitation of the same tryptic peptide showed that hydroxylation was essentially complete, within the limits of detection (>95%; Figure 3B). Similar results were obtained with endogenous eRF1 purified from HeLa, A549, and U2OS cells (Figures 3B and 3C). To determine whether endogenous eRF1 K63 hydroxylation requires Jmjd4, we suppressed the expression of the endogenous enzyme by siRNA. Knockdown of Jmjd4 expression substantially inhibited the hydroxylation of newly synthesized eRF1 (Figures 3C and 3D), suggesting that other lysyl hydroxylases are unlikely to contribute to its hydroxylation. To test whether these effects were due to reduced Jmjd4 activity, we transfected Jmjd4 siRNA into cells that ectopically express Jmjd4 siRNA-resistant mRNAs, which were either wild-type or inactive (Figures 3C and 3D). Expression of hemagglutinin (HA)-Jmjd4 successfully restored eRF1 hydroxylation, whereas HA-Jmjd4 H189A did not. Taken together, these data indicate that the abundant and ubiquitous hydroxylation of endogenous eRF1 in tissue culture cell lines is dependent on Jmjd4 catalytic activity. To determine whether eRF1 K63 hydroxylation is a physiologically relevant modification, we next purified eRF1 from a variety of mammalian tissues. Importantly, LC-MS analyses of eRF1 purified from several mouse tissues and rabbit reticulocyte lysate indicated that eRF1 hydroxylation is abundant (>90%) and conserved (Figure 3E).

### Endogenous eRF1 K63 Hydroxylation Is Dependent on 2-Oxoglutarate and Oxygen

Consistent with the requirement of eRF1 K63 hydroxylation for Jmjd4 activity, treatment of HeLa, U2OS, and Hep3B cells with a cell permeable form of the 2OG competitor NOG (dimethyloxalylglycine; DMOG) also reduced the hydroxylation of newly synthesized eRF1 (Figure S3B and data not shown). 2OG oxygenases also depend on molecular oxygen to create a hydroxyl group in the prime substrate. To test the sensitivity of eRF1 hydroxylation to oxygen availability, we incubated HeLa, U2OS, and Hep3B cells in normoxia (21% O_2_), hypoxia (1% and 0.1% O_2_), and anoxia (0% O_2_). MS analyses of eRF1 synthesized under these conditions confirmed that K63 hydroxylation was reduced by profound hypoxia (Figure S3C and data not shown). However, Jmjd4 retains substantial activity even under severe hypoxia (≤1% O_2_), similar to some related 2OG oxygenases (Ge et al., 2012; Tian et al., 2011), suggesting that it is unlikely to act as an oxygen sensor analogous to the HIF hydroxylases (Kaelin and Ratcliffe, 2008).

### Jmjd4 Activity Is Required for Translational Termination

**Figure 4:**
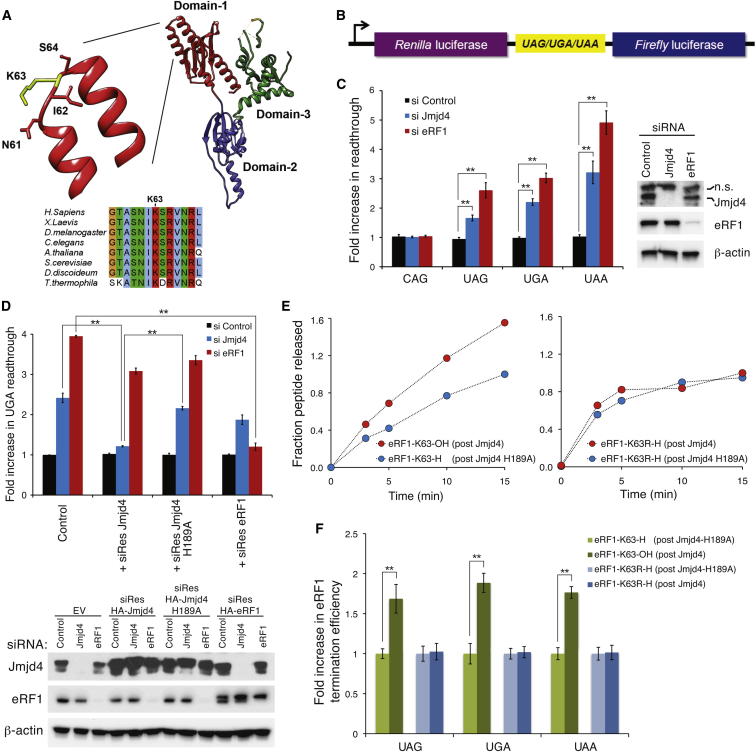
eRF1 K63 Hydroxylation Promotes Translational Termination Efficiency (A) Top: K63 is located at the apex of an α-helical extension within eRF1 domain 1 (Chimera and Protein Data Bank ID 1DT9) (Song et al., 2000). Bottom: alignment of the eRF1 NIKS region, indicating complete conservation of K63 across diverse species (Jalview). (B) A bicistronic reporter for measuring stop codon readthrough (Grentzmann et al., 1998) with luciferase cDNAs separated by a stop or sense codon (CAG) within a leaky termination sequence from tobacco mosaic virus. (C) Jmjd4 knockdown promotes stop codon readthrough. Left: HeLa cells were transfected with the reporters in (B) prior to siRNA (si) and dual luciferase assay. Note that K63 hydroxylation is ∼50% under similar knockdown conditions (see Figure 3 legend). Data represent mean ± SEM. Statistical significance was evaluated by ANOVA followed by Dunett’s post hoc test comparing Jmjd4 and eRF1 knockdown samples to control (^∗∗^p < 0.01). Right: immunoblot of cell extracts (n.s., nonspecific). (D) Jmjd4 activity is required for efficient translational termination. Top: stop codon readthrough assays were performed as in (C) using HeLa cell lines expressing empty vector, siRes-HA-Jmjd4, siRes-HA-Jmjd4 H189A, or siRes-HA-eRF1 mRNAs. Bottom: immunoblot of cell extracts. Data represent mean ± SEM. Comparisons across cell lines were made by ANOVA followed by Bonferroni post hoc test (^∗∗^p < 0.01). (E) K63 hydroxylation increases the catalytic efficiency of eRF1 in vitro. Unhydroxylated (post-Jmjd4-H189A treatment; blue) or hydroxylated (post-Jmjd4 treatment; red; ∼60% hydroxylation) wild-type (left) or K63R (right) eRF1 were added to pretermination ribosomal complexes before quantifying released radiolabelled peptides over time (shown is an example of data obtained with the UAA stop codon). Note that contaminating Jmjd4 was removed from eRF1 preparations (see Figure S4N). (F) Termination efficiencies (K_cat_/K_M_ × 10^4^ [M^−1^s^−1^]) of wild-type (green) and K63R eRF1 (blue) at all three stop codons were calculated from initial rates and plotted as fold increase relative to Jmjd4 H189A-treated eRF1. Data represent mean ± SEM. Statistical significance was evaluated by paired two-tailed Student’s t test (^∗∗^p < 0.01). See also Figure S4.

Termination of eukaryotic protein translation is mediated by eRF1, the guanosine triphosphatase (GTPase) eRF3, and the ATPase ABCE1 (Kisselev et al., 2003; Shoemaker and Green, 2011). eRF1 consists of three functional domains: domain 1 decodes stop codons, domain 2 facilitates peptidyl-tRNA hydrolysis, and domain 3 recruits eRF3 (Figure 4A) (Nakamura and Ito, 2011). Consistent with a role in stop codon recognition, a cryoelectron microscopy (cryo-EM) structure of the ribosomal pretermination complex places domain 1 deep within the decoding center of the 40S subunit (Taylor et al., 2012). Functional studies have implicated highly conserved motifs in domain 1 (e.g., GTX, YXCXXXF, and NIKS) (Figure 4A) in stop codon recognition and its coupling to peptidyl-tRNA hydrolysis (Bertram et al., 2000; Conard et al., 2012; Fan-Minogue et al., 2008; Frolova et al., 2002; Song et al., 2000). Crosslinking experiments suggest that under some conditions the lysine within the NIKS motif contacts the uridine nucleotide in the first position of a stop codon (Chavatte et al., 2002). Although the precise molecular function of the NIKS motif is under debate, it is known to play an important role in translational termination (Bertram et al., 2000; Conard et al., 2012; Frolova et al., 2002; Kisselev et al., 2003; Nakamura and Ito, 2011; Song et al., 2000). Critically, the lysine within this motif (K63; Figure 4A) is the residue hydroxylated by Jmjd4 to >90% in the steady state. Therefore, we hypothesized that K63 hydroxylation may promote eRF1 function and that its inhibition could cause stop codon readthrough. To test this, we used established stop codon readthrough reporters consisting of tandem in-frame *Renilla* and firefly luciferase cDNAs separated by each stop codon (*Renilla*:*stop*:firefly) (Figure 4B) (Grentzmann et al., 1998). Stop codon readthrough is indicated by an increase in firefly relative to *Renilla* luciferase activities. Importantly, knockdown of Jmjd4 or eRF1 in HeLa, U2OS, or Hep3B cells induced readthrough of all three stop codons embedded within a “leaky” termination sequence (Figures 4C, S4A, and S4B). These effects were apparently specific to termination and translation, as they were not observed with sequences lacking a stop codon (*Renilla*:*CAG*:firefly) (Figures 4C, S4A, and S4B) and were not associated with aberrant transcript splicing or changes in abundance of firefly relative to *Renilla* luciferase mRNAs (Figures S4C–S4E) (Holcik et al., 2005; Lemp et al., 2012). Furthermore, these effects were specific to Jmjd4 (at least among closely related hydroxylases), since siRNA knockdown of FIH, MINA53, and Jmjd6 did not induce stop codon readthrough (Figure S4F).

To further explore the specificity and generality of these results, we tested an independent stop codon reporter (Figure S4G) in an additional cell type: Jmjd4 or eRF1 siRNA in A549 cells also increased readthrough of a stop codon within a leaky termination sequence when using a β-galactosidase:*stop*:firefly reporter (Figures S4H and S4I, respectively). Importantly, similar results were also obtained using the same reporter with a stop codon embedded in a “strong” termination sequence (Figure S4J). Taken together, the results presented here suggest that Jmjd4 may have a widespread role in translational termination, perhaps consistent with ubiquitous and abundant eRF1 K63 hydroxylation (Figure 3).

To determine whether the role of Jmjd4 in translational termination depends on its hydroxylase activity, we repeated knockdowns in cells expressing siRNA-resistant wild-type or inactive Jmjd4 mRNAs. Jmjd4 siRNA induced stop codon readthrough in inactive HA-Jmjd4 cells (*Renilla*:*stop*:firefly reporter), whereas cells expressing wild-type HA-Jmjd4 retained normal translational termination (Figure 4D) consistent with restored eRF1 hydroxylation (Figure 3C). As expected, eRF1 siRNA was still sufficient for stop codon readthrough in cells expressing siRNA-resistant Jmjd4 mRNA, but not in those expressing siRNA-resistant eRF1 (Figure 4D). Similar results demonstrating a requirement for Jmjd4 activity were obtained with the β-galactosidase:*stop*:firefly reporter using either the leaky or strong termination sequence (Figures S4K–S4M). Together, these data indicate that Jmjd4 catalysis promotes translational termination efficiency.

### K63 Hydroxylation Promotes the Translational Termination Efficiency of eRF1 In Vitro

Next, we sought to determine whether the role of Jmjd4 activity in translational termination in cells is a direct consequence of eRF1 K63 hydroxylation. To this end, we used a fully reconstituted in vitro translation system where eRF1 activity is measured by the release of translated peptides from stalled pretermination complexes (Alkalaeva et al., 2006). Consistent with previous reports (Alkalaeva et al., 2006; Kryuchkova et al., 2013), unhydroxylated recombinant eRF1 exhibited substantial catalytic efficiency in this assay (1 − 2 K_cat_/K_M_ × 10^4^ [M^−1^s^−1^]). Importantly, however, partial K63 hydroxylation (∼60%; data not shown) further increased eRF1 termination efficiency at all three stop codons (Figures 4E, 4F, and S4N). In contrast, an eRF1 K63R mutant failed to show any increase in activity following incubation with Jmjd4, thereby assigning the effect of Jmjd4 on translation termination to hydroxylation of K63.

## Discussion

The work presented here describes the regulatory modification of a highly conserved region of eRF1 known to play an important role in translational termination (Bertram et al., 2000; Conard et al., 2012; Fan-Minogue et al., 2008; Frolova et al., 2002; Song et al., 2000). We have shown that hydroxylation of the eRF1 NIKS motif promotes polypeptide release from pretermination ribosomal complexes in vitro and that reduced Jmjd4 activity causes stop codon readthrough in vivo (Figure 4). Previously known posttranslational modifications of eRF1 are limited to phosphorylation of domain 3 (Kallmeyer et al., 2006) and *N*^5^-glutamine methylation of a GGQ motif within domain 2 required for peptidyl-tRNA hydrolysis (Graille et al., 2012). In contrast to the consistent effects of Jmjd4-dependent NIKS hydroxylation (Figure 4), the effect of these modifications on eukaryotic translational termination is unclear.

A general role for K63 hydroxylation during a fundamental step in translational termination may be consistent with the abundance and ubiquity of eRF1 hydroxylation (Figure 3), and the requirement for Jmjd4 activity observed across multiple experimental models, cell types and stop codon contexts (Figures 4 and S4). Hydroxylation can modulate protein function via intra- or intermolecular hydrogen-bonds or electronic effects (Coleman et al., 2007; Hon et al., 2002; Loenarz and Schofield, 2011). Recent studies suggest that the loop containing the NIKS motif and the helical extension surrounding it have some inherent plasticity (Polshakov et al., 2012). An interaction between hydroxylated-K63 and a neighboring eRF1 residue could modulate this flexibility and optimize the conformation of this domain during stop codon recognition and/or subsequent steps of termination. Alternatively, K63 hydroxylation could mediate an interaction with the stop codon Uridine (Chavatte et al., 2002), ribosome or other molecules involved in the termination process. The exact molecular mechanism by which eRF1 K63 hydroxylation regulates translational termination is unclear, and will be the subject of future investigation.

The completeness of K63 hydroxylation may argue against a switch-like function in normal tissues. Rather, the fact that Jmjd4 requires key nutrients for activity raises the possibility that nutrient stress, pathological conditions or pharmacological intervention could promote stop codon readthrough via inhibition of eRF1 hydroxylation. Regulated stop codon readthrough may provide a means of controlling eukaryotic gene expression (Steneberg et al., 1998; Yamaguchi et al., 2012). Furthermore, pharmacological readthrough of premature termination codons is attracting interest as a therapeutic approach for treating diseases caused by nonsense mutations (Bidou et al., 2012). The possibility that targeting Jmjd4 could promote the action of these agents is of interest.

Finally, our study provides insights into the expanding biology of 2OG oxygenases. Previous examples of lysyl-hydroxylation are restricted to the C5 position. Our results demonstrate that 2OG oxygenases can also hydroxylate at C4 (Figure 2). This raises the possibility that other forms of lysyl-hydroxylation also exist, possibly catalyzed by related but as yet unassigned Jmjd proteins. Together with recently identified hydroxylations of ribosomal proteins (Ge et al., 2012) and tRNA anticodons (Fu et al., 2010; Noma et al., 2010; van den Born et al., 2011) the work also suggests that protein translation, and particularly ‘decoding’, may be a major target of 2OG oxygenases.

## Experimental Procedures

Plasmid construction and culture, transfection, and treatment of cells are described in the Supplemental Experimental Procedures. Immunoblotting was performed as previously described (Ge et al., 2012). Recombinant human eRF1 was purified as described (Frolova et al., 2002). Human recombinant Jmjd4 was purified from *E. coli* lysates using Ni-NTA resin (QIAGEN) following standard procedures. For reporter assays, siRNA-treated cells were transfected with reporter vectors for 48 hr before lysis in passive lysis buffer (Promega). Samples were freeze thawed before measuring luciferase activities using the Dual-Luciferase System (Promega). For the β-galactosidase:*stop*:firefly vector, luciferase was assayed using luciferin (Promega) and β-galactosidase with the FluorAce Kit (Bio-Rad). All assays were performed on a Safire2 Microplate Reader (Tecan). Experimental procedures used for mouse tissue analysis passed ethical review by the Medical Sciences divisional Local Ethical Review panel (Oxford University) and were performed under UK Home Office regulations (Animal [Scientific Procedures] Act 1986). Details of the remaining methods are provided in the Supplemental Experimental Procedures.

## Supplemental Information

Document S1. Supplemental Experimental Procedures and Figures S1–S4

## References

[bib1] Alkalaeva E.Z., Pisarev A.V., Frolova L.Y., Kisselev L.L., Pestova T.V. (2006). In vitro reconstitution of eukaryotic translation reveals cooperativity between release factors eRF1 and eRF3. Cell.

[bib2] Bertram G., Bell H.A., Ritchie D.W., Fullerton G., Stansfield I. (2000). Terminating eukaryote translation: domain 1 of release factor eRF1 functions in stop codon recognition. RNA.

[bib3] Bidou L., Allamand V., Rousset J.P., Namy O. (2012). Sense from nonsense: therapies for premature stop codon diseases. Trends Mol. Med..

[bib4] Chavatte L., Seit-Nebi A., Dubovaya V., Favre A. (2002). The invariant uridine of stop codons contacts the conserved NIKSR loop of human eRF1 in the ribosome. EMBO J..

[bib5] Coleman M.L., Ratcliffe P.J. (2009). Signalling cross talk of the HIF system: involvement of the FIH protein. Curr. Pharm. Des..

[bib6] Coleman M.L., McDonough M.A., Hewitson K.S., Coles C., Mecinovic J., Edelmann M., Cook K.M., Cockman M.E., Lancaster D.E., Kessler B.M. (2007). Asparaginyl hydroxylation of the Notch ankyrin repeat domain by factor inhibiting hypoxia-inducible factor. J. Biol. Chem..

[bib7] Conard S.E., Buckley J., Dang M., Bedwell G.J., Carter R.L., Khass M., Bedwell D.M. (2012). Identification of eRF1 residues that play critical and complementary roles in stop codon recognition. RNA.

[bib8] Fan-Minogue H., Du M., Pisarev A.V., Kallmeyer A.K., Salas-Marco J., Keeling K.M., Thompson S.R., Pestova T.V., Bedwell D.M. (2008). Distinct eRF3 requirements suggest alternate eRF1 conformations mediate peptide release during eukaryotic translation termination. Mol. Cell.

[bib9] Frolova L., Seit-Nebi A., Kisselev L. (2002). Highly conserved NIKS tetrapeptide is functionally essential in eukaryotic translation termination factor eRF1. RNA.

[bib10] Fu Y., Dai Q., Zhang W., Ren J., Pan T., He C. (2010). The AlkB domain of mammalian ABH8 catalyzes hydroxylation of 5-methoxycarbonylmethyluridine at the wobble position of tRNA. Angew. Chem. Int. Ed. Engl..

[bib11] Ge W., Wolf A., Feng T., Ho C.H., Sekirnik R., Zayer A., Granatino N., Cockman M.E., Loenarz C., Loik N.D. (2012). Oxygenase-catalyzed ribosome hydroxylation occurs in prokaryotes and humans. Nat. Chem. Biol..

[bib12] Graille M., Figaro S., Kervestin S., Buckingham R.H., Liger D., Heurgué-Hamard V. (2012). Methylation of class I translation termination factors: structural and functional aspects. Biochimie.

[bib13] Grentzmann G., Ingram J.A., Kelly P.J., Gesteland R.F., Atkins J.F. (1998). A dual-luciferase reporter system for studying recoding signals. RNA.

[bib14] Holcik M., Graber T., Lewis S.M., Lefebvre C.A., Lacasse E., Baird S. (2005). Spurious splicing within the XIAP 5′ UTR occurs in the Rluc/Fluc but not the betagal/CAT bicistronic reporter system. RNA.

[bib15] Hon W.C., Wilson M.I., Harlos K., Claridge T.D., Schofield C.J., Pugh C.W., Maxwell P.H., Ratcliffe P.J., Stuart D.I., Jones E.Y. (2002). Structural basis for the recognition of hydroxyproline in HIF-1 alpha by pVHL. Nature.

[bib16] Kaelin W.G., Ratcliffe P.J. (2008). Oxygen sensing by metazoans: the central role of the HIF hydroxylase pathway. Mol. Cell.

[bib17] Kallmeyer A.K., Keeling K.M., Bedwell D.M. (2006). Eukaryotic release factor 1 phosphorylation by CK2 protein kinase is dynamic but has little effect on the efficiency of translation termination in Saccharomyces cerevisiae. Eukaryot. Cell.

[bib18] Kisselev L.L., Buckingham R.H. (2000). Translational termination comes of age. Trends Biochem. Sci..

[bib19] Kisselev L., Ehrenberg M., Frolova L. (2003). Termination of translation: interplay of mRNA, rRNAs and release factors?. EMBO J..

[bib20] Klose R.J., Kallin E.M., Zhang Y. (2006). JmjC-domain-containing proteins and histone demethylation. Nat. Rev. Genet..

[bib21] Kryuchkova P., Grishin A., Eliseev B., Karyagina A., Frolova L., Alkalaeva E. (2013). Two-step model of stop codon recognition by eukaryotic release factor eRF1. Nucleic Acids Res..

[bib22] Lemp N.A., Hiraoka K., Kasahara N., Logg C.R. (2012). Cryptic transcripts from a ubiquitous plasmid origin of replication confound tests for cis-regulatory function. Nucleic Acids Res..

[bib23] Loenarz C., Schofield C.J. (2008). Expanding chemical biology of 2-oxoglutarate oxygenases. Nat. Chem. Biol..

[bib24] Loenarz C., Schofield C.J. (2011). Physiological and biochemical aspects of hydroxylations and demethylations catalyzed by human 2-oxoglutarate oxygenases. Trends Biochem. Sci..

[bib25] Mantri M., Krojer T., Bagg E.A., Webby C.J., Butler D.S., Kochan G., Kavanagh K.L., Oppermann U., McDonough M.A., Schofield C.J. (2010). Crystal structure of the 2-oxoglutarate- and Fe(II)-dependent lysyl hydroxylase JMJD6. J. Mol. Biol..

[bib26] Nakamura Y., Ito K. (2011). tRNA mimicry in translation termination and beyond. Wiley Interdiscip Rev RNA.

[bib27] Noma A., Ishitani R., Kato M., Nagao A., Nureki O., Suzuki T. (2010). Expanding role of the jumonji C domain as an RNA hydroxylase. J. Biol. Chem..

[bib28] Polshakov V.I., Eliseev B.D., Birdsall B., Frolova L.Y. (2012). Structure and dynamics in solution of the stop codon decoding N-terminal domain of the human polypeptide chain release factor eRF1. Protein Sci..

[bib29] Shoemaker C.J., Green R. (2011). Kinetic analysis reveals the ordered coupling of translation termination and ribosome recycling in yeast. Proc. Natl. Acad. Sci. USA.

[bib30] Song H., Mugnier P., Das A.K., Webb H.M., Evans D.R., Tuite M.F., Hemmings B.A., Barford D. (2000). The crystal structure of human eukaryotic release factor eRF1—mechanism of stop codon recognition and peptidyl-tRNA hydrolysis. Cell.

[bib31] Steneberg P., Englund C., Kronhamn J., Weaver T.A., Samakovlis C. (1998). Translational readthrough in the hdc mRNA generates a novel branching inhibitor in the Drosophila trachea. Genes Dev..

[bib32] Taylor D., Unbehaun A., Li W., Das S., Lei J., Liao H.Y., Grassucci R.A., Pestova T.V., Frank J. (2012). Cryo-EM structure of the mammalian eukaryotic release factor eRF1-eRF3-associated termination complex. Proc. Natl. Acad. Sci. USA.

[bib33] Tian Y.M., Yeoh K.K., Lee M.K., Eriksson T., Kessler B.M., Kramer H.B., Edelmann M.J., Willam C., Pugh C.W., Schofield C.J., Ratcliffe P.J. (2011). Differential sensitivity of hypoxia inducible factor hydroxylation sites to hypoxia and hydroxylase inhibitors. J. Biol. Chem..

[bib34] Unoki M., Masuda A., Dohmae N., Arita K., Yoshimatsu M., Iwai Y., Fukui Y., Ueda K., Hamamoto R., Shirakawa M. (2013). Lysyl 5-hydroxylation, a novel histone modification, by Jumonji domain containing 6 (JMJD6). J. Biol. Chem..

[bib35] van den Born E., Vågbø C.B., Songe-Møller L., Leihne V., Lien G.F., Leszczynska G., Malkiewicz A., Krokan H.E., Kirpekar F., Klungland A., Falnes P.Ø. (2011). ALKBH8-mediated formation of a novel diastereomeric pair of wobble nucleosides in mammalian tRNA. Nat Commun.

[bib36] Walsh C.T. (2005). Posttranslational Modification of Proteins: Expanding Nature’s Inventory.

[bib37] Webby C.J., Wolf A., Gromak N., Dreger M., Kramer H., Kessler B., Nielsen M.L., Schmitz C., Butler D.S., Yates J.R. (2009). Jmjd6 catalyses lysyl-hydroxylation of U2AF65, a protein associated with RNA splicing. Science.

[bib38] Yamaguchi Y., Hayashi A., Campagnoni C.W., Kimura A., Inuzuka T., Baba H. (2012). L-MPZ, a novel isoform of myelin P0, is produced by stop codon readthrough. J. Biol. Chem..

