# Supplementary material for: Optimal Translational Termination Requires C4 Lysyl Hydroxylation of eRF1
Source: Mol Cell. 2014 Feb 20;53(4):645–54. doi: 10.1016/j.molcel.2013.12.028 (PMC3991326; doi:10.1016/j.molcel.2013.12.028)
Supplement: Document S1. Supplemental Experimental Procedures and Figures S1–S4 [file mmc1.pdf]

Molecular Cell, Volume *53*

Supplemental Information

Optimal Translational Termination Requires C4 Lysyl Hydroxylation of eRF1

Tianshu Feng, Atsushi Yamamoto, Sarah E. Wilkins, Elizaveta Sokolova, Luke A. Yates, Martin Münzel, Pooja Singh, Richard J. Hopkinson, Roman Fischer, Matthew E. Cockman, Jake Shelley, David C. Trudgian, Johannes Schödel, James S. O. McCullagh, Wei Ge, Benedikt M. Kessler, Robert J. Gilbert, Ludmila Y. Frolova, Elena Alkalaeva, Peter J. Ratcliffe, Christopher J. Schofield, and Mathew L. Coleman

Inventory of Supplemental Information:

Figure S1, related to Figure 1

Phylogenetic tree of the catalytic domains of all known JmjC domain proteins.

MS/MS spectrum demonstrating K63 hydroxylation of overexpressed eRF1.

Figure S2, related to Figure 2

Amino acid analyses of an eRF1 K63 hydroxylated cyclic peptide demonstrates Jmjd4 is a C4 lysyl hydroxylase.

Figure S3, related to Figure 3

MS/MS spectrum of Arg-C digested endogenous eRF1 confirms K63 hydroxylation.

Endogenous eRF1 K63 hydroxylation is inhibited by 2OG competitive inhibitors and hypoxia.

Figure S4, related to Figure 4

Jmjd4 catalysis regulates translational termination in multiple cell types and sequence contexts.

Supplemental Experimental Procedures

Supplemental References

Figure S1

A

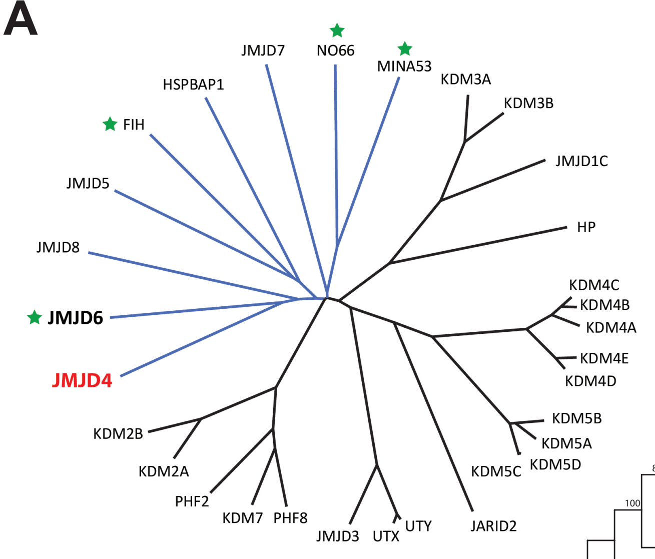

B

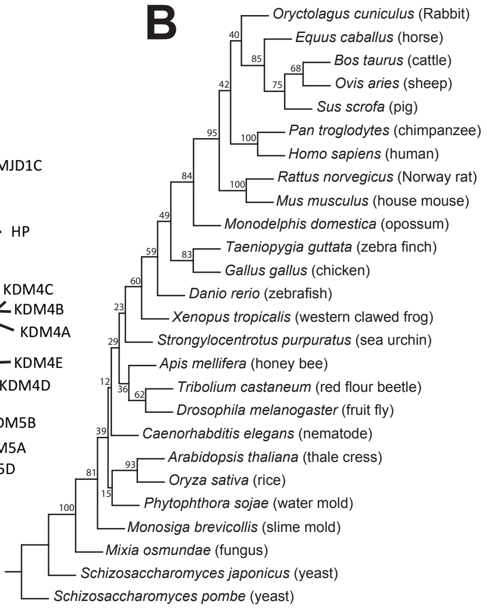

C

Description	Localisation	PSMs	Peptides	% Coverage
eRF1	Cytoplasmic	213	33	75.1
eRF3A	Cytoplasmic	86	32	56.3
FAM40A	Nuclear	6	3	5.1
GGCX Vitamin K-dependent gamma-carboxylase	ER membrane	5	3	2.5
Transcription elongation factor A protein-like 4	Nuclear	5	3	15.1
DCTN1	Cytoplasmic	3	4	5.4
PARP4 Poly [ADP-ribose] polymerase 4	Cytoplasmic/Nuclear	3	3	2.4
ZNF180 Zinc finger protein 180	Nuclear	3	2	9

D

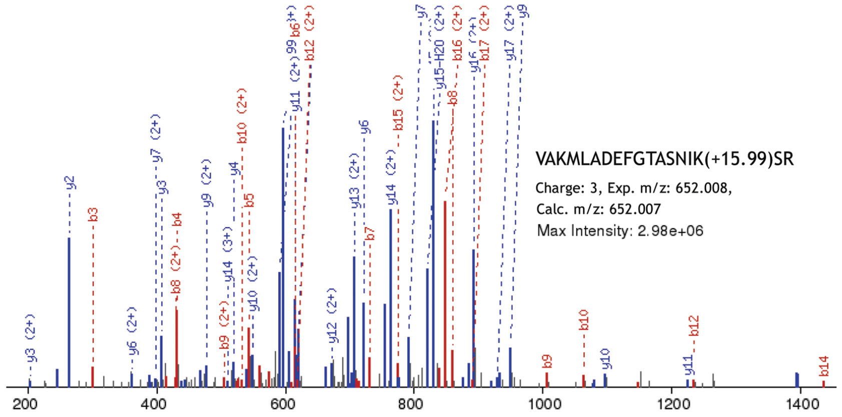

Sequence Ladder Ions

Key: m/z out of range of spectrum | matched c-term ion | unmatched c-term ion | matched n-term ion | unmatched n-term ion

	V	A	K	M	L	A	D	E	F	G	T	A	S	N	I	K	S	R	
	1	2	3	4	5	6	7	8	9	10	11	12	13	14	15	16	17	18	
a		143.12																	a
b		171.11	299.21	430.25	543.33	614.37	729.40	858.44	1005.51	1062.53	1163.58	1234.61	1321.65	1435.69	1548.77	1692.86	1779.89		b
b (2+)			150.11	215.63	272.17	307.69	365.20	429.72	503.26	531.77	582.29	617.81	661.33	718.35	774.89	846.93	890.45		b (2+)
b (3+)																564.96	593.97		b (3+)
y		1854.94	1783.90	1655.81	1524.77	1411.68	1340.64	1225.62	1096.57	949.51	892.48	791.44	720.40	633.37	519.32	406.24	262.15	175.12	y
y (2+)		927.97	892.45	828.41	762.89	706.34	670.83	613.31	548.79	475.26	446.75	396.22	360.70	317.19	260.17	203.62	131.58	88.06	y (2+)
y (3+)		618.98	595.31	552.61	508.93	471.23	447.55	409.21	366.20	317.17	298.17	264.48	240.80	211.79	173.78	136.09			y (3+)
	V	A	K	M	L	A	D	E	F	G	T	A	S	N	I	K	S	R	
	18	17	16	15	14	13	12	11	10	9	8	7	6	5	4	3	2	1	

Figure S1, related to Figure 1. (**A**) BLAST analysis of the NCBI non-redundant protein database was used to identify Jmjd4 proteins from the indicated 26 eukaryotic species (orthologues of Jmjd4 were not found in bacteria or archaea). A multiple sequence alignment of the JmjC domains was carried out using Clustal Omega, and a phylogenetic tree constructed with PHYLIP using bootstrap (seqboot) and maximum likelihood (protml) methods. Bootstrap values are listed above the internodes, and represent the percentage of sampled trees used in the analysis that contained the consensus partition. NCBI reference IDs: *H. sapiens* (NP_075383), *P. troglodytes* (XP_514250), *M. musculus* (NP_848774), *R. norvegicus* (NP_001099254), *B. Taurus* (XP_582558), *O. aries* (XP_004009182), *S. scrofa* (XP_003123657), *M. domestica* (XP_001376273), *O. cuniculus* (XP_002723947), *E. caballus* (XP_005599806), *G. gallus* (NP_001026130), *T. guttata* (XP_002194504), *D. rerio* (NP_001070096), *X. tropicalis* (XP_002939408), *T. castaneum* (XP_974646), *D. melanogaster* (NP_609870), *A. mellifera* (XP_395655), *S. purpuratus* (XP_001181165), *C. elegans* (NP_001255071), *A. thaliana* (NP_201113), *O. sativa* (NP_001043285), *S. pombe* (NP_593806), *S. japonicas* (EEB07354), *M. osmundae* (GAA97942), *M. brevicollis* (XP_001750327), *P. sojae* (EGZ28846). It was not possible to identify clear orthologues of Jmjd4 (or Jmjd6) in *S. cerevisiae* using this approach. (**B**) A phylogenetic tree was constructed using PHYLIP (Phylogeny Inference Package) from a Clustal Omega protein sequence alignment of the JmjC domains of all known human JmjC-domain containing proteins. The branches corresponding to ‘JmjC-only’ proteins are highlighted in blue. Enzymes of this sub-family with an identified hydroxylase function are indicated by green stars. (**C**) Summary of proteins interacting with Jmjd4 in an activity-dependent manner. Proteomic pulldown MS data from empty vector, FLAG-Jmjd4, and FLAG-Jmjd4 H189A (inactive) were cross-referenced and then filtered to highlight those proteins only identified in active Jmjd4 complexes. These candidate substrates were ranked according to MS parameters

that are proportional to abundance in the sample, including peptide spectral matches (PSMs), the number of peptides detected, and the percentage sequence coverage of the protein. eRF1 and eRF3a were the only two proteins that demonstrated significant activity-dependent abundance. The cellular localization of each candidate is indicated (where known) for comparison to Jmjd4 (Figure 1). (**D**) MS/MS spectrum of a peptide containing residues 48-65 derived from Arg-C digested overexpressed eRF1 indicating hydroxylation of K63. Upper panel: MS/MS spectra showing the y- (blue) and b-ion (red) series for singly, doubly (2+) and triply (3+) charged fragments. Lower panel: table of the corresponding matched y- and b-ion fragments indicating the masses detected.

Figure S2

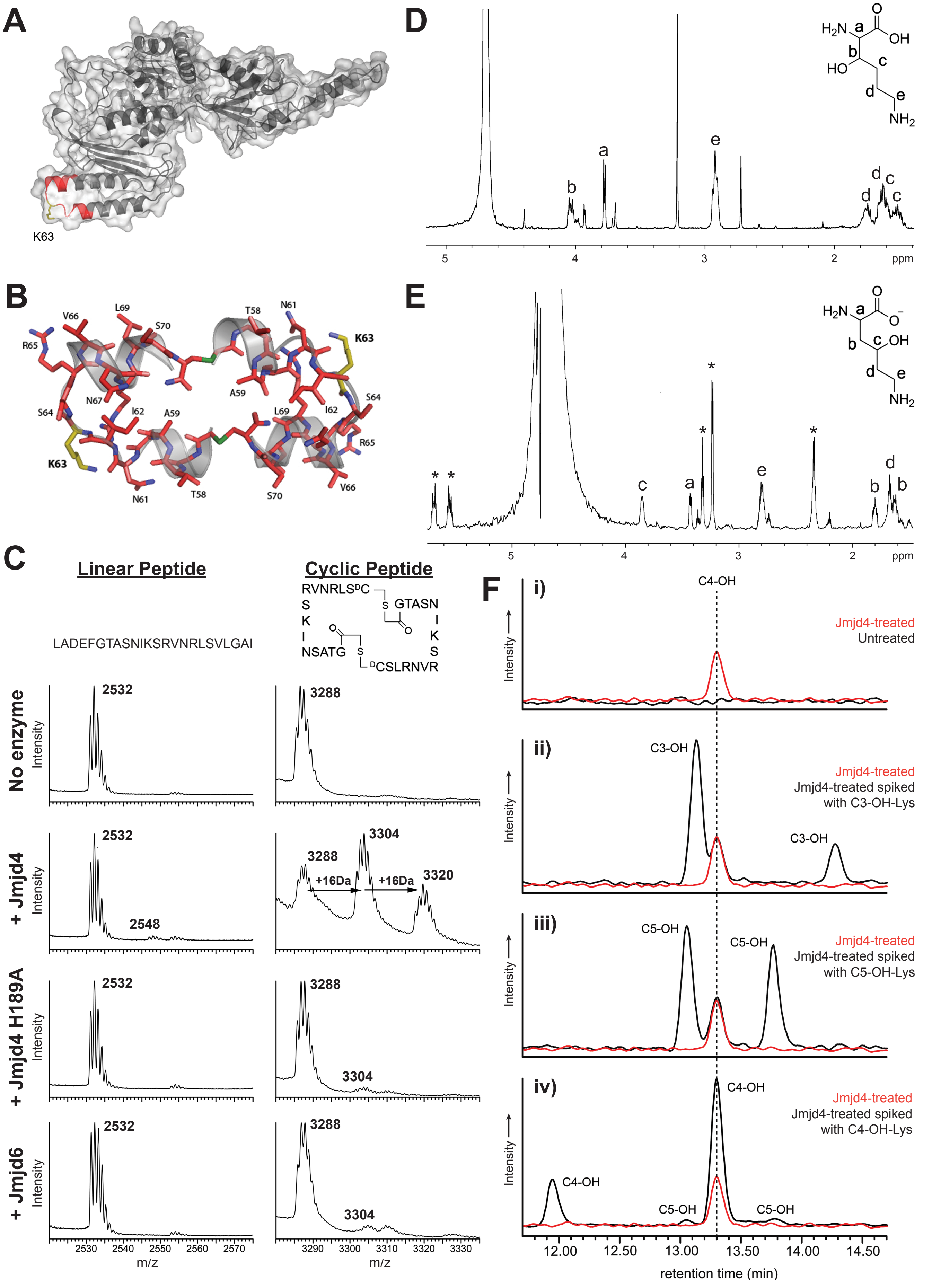

A
K63
B
V66
L69
S70
T58
N61
K63
R65
N67
A59
S64
I62
A59
L69
I62
S64
R65
K63
N61
T58
S70
V66
C
Linear Peptide
Cyclic Peptide
LADEFGTASNIKSRVNRLSVLGAI
RVNRLS^DC
S GTASN
S
K
I
K
I
S
NSATG S
^DCSLRNVR
No enzyme
+ Jmjd4
+ Jmjd4 H189A
+ Jmjd6
Intensity
2532
3288
2548
3304
3320
+16Da
m/z
D
E
H2N
OH
HO
NH2
a
b
c
d
e
*
ppm
F
i)
ii)
iii)
iv)
C4-OH
C3-OH
C5-OH
Jmjd4-treated
Untreated
Jmjd4-treated spiked with C3-OH-Lys
Jmjd4-treated spiked with C5-OH-Lys
Jmjd4-treated spiked with C4-OH-Lys
retention time (min)

Figure S2, related to Figure 2. A cyclic eRF1 peptide supports Jmjd4-dependent C4 lysyl hydroxylation. (**A**) Crystal structure of eRF1 highlighting the position of K63 (*yellow*) at the apex (*red*) of an α-helical extension within domain-1. (**B**) Overlay of the cyclic dimer sequence (red ball and stick) with its predicted secondary structure (grey). Note the similarity in predicted conformation to the native structure above. **(C)** Jmjd4 efficiently hydroxylates a cyclic eRF1 peptide *in vitro*. Linear (left column) or cyclic (right column) eRF1 peptides were incubated with cofactors alone (no enzyme), wildtype or inactive (H189A) recombinant Jmjd4, or Jmjd6 prior to MALDI-TOF MS analyses to quantify the extent of hydroxylation (indicated by an enzyme-dependent +16Da mass shift). Note that the cyclic peptide shows two +16Da shifts, consistent with hydroxylation of both lysyl residues within the cyclic peptide. In contrast, incubation of the linear peptide leads to a minor +16Da peak (2548Da). Although dependent on Jmjd4 activity we were unable to definitively assign this peak as arising from K63 hydroxylation by MS/MS or amino acid analysis due to insufficient conversion. (**D**) NMR assignment of a mixture of stereoisomers of 3-hydroxylysine. ^{1}H NMR spectrum of the reaction mixture after treatment with H_2SO_4. Resonances corresponding to 3-hydroxylysine (a-e) are highlighted. (**E**) NMR assignment of a mixture of stereoisomers of 4-hydroxylysine. ^{1}H NMR spectrum of the reaction mixture after treatment with H_2SO_4. Resonances corresponding to 4-hydroxylysine (a-e) and the starting material (4,5-dehydrolysine, asterisks) are highlighted. (**F**) Amino acid analysis provides evidence for Jmjd4-dependent C4-lysyl hydroxylation. (i) LC-MS analysis of a Jmjd4-reacted cyclic eRF1 peptide following enzymatic digestion indicates a single major product (red). Spiking the sample with known hydroxylysine standards including C3-hydroxylysine (ii), C5-hydroxylysine (iii) or a mixture of C4- and C5-hydroxylysine (iv) assigns the peak as C4-hydroxylysine. Two peaks are observed per standard because each is a mixture of stereoisomers.

Figure S3

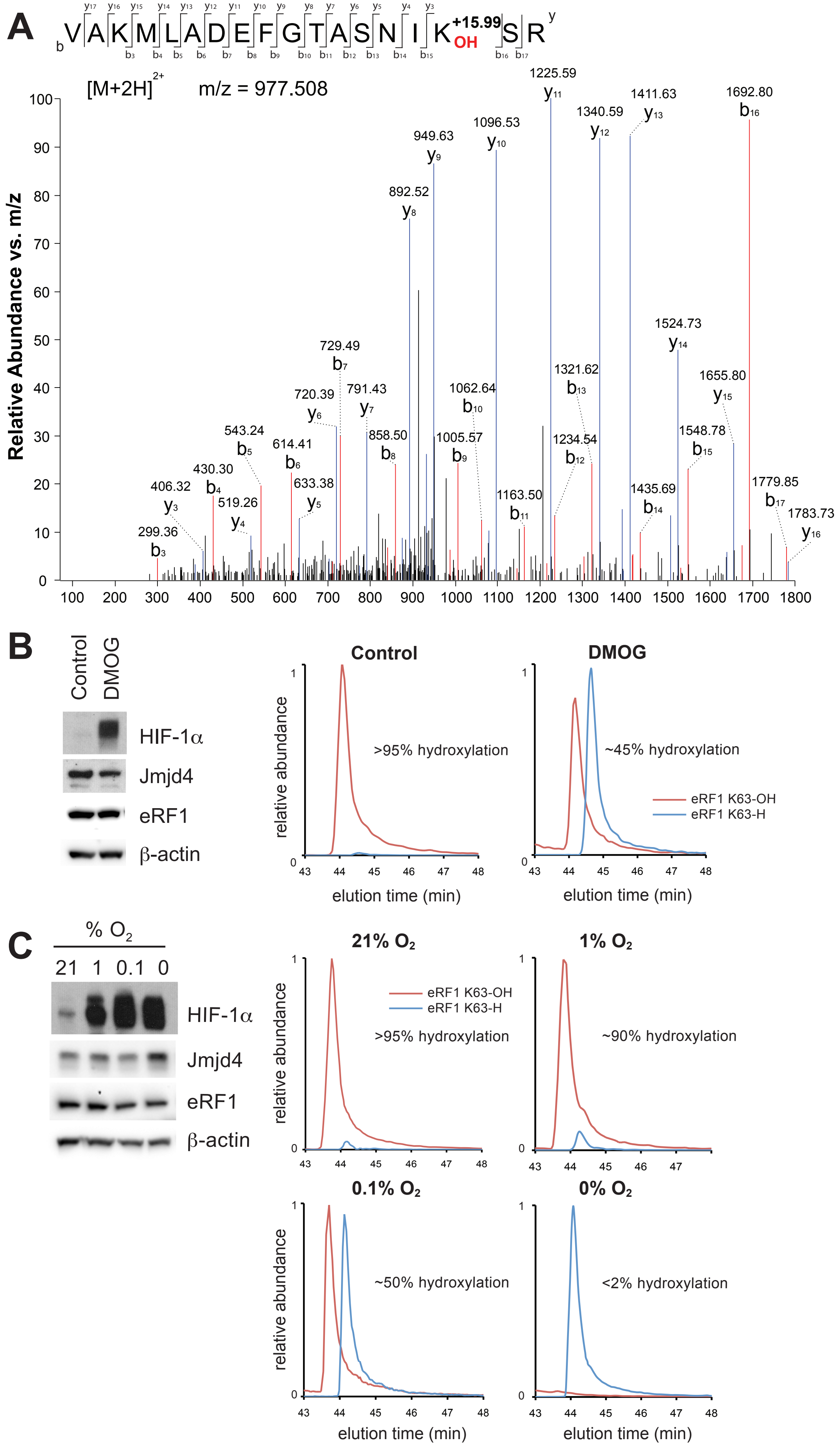
A
VAKMLADEFGTASNIK+15.99 OH SR
[M+2H]2+ m/z = 977.508
Relative Abundance vs. m/z
B
Control
DMOG
HIF-1α
Jmjd4
eRF1
β-actin
Control
DMOG
>95% hydroxylation
~45% hydroxylation
eRF1 K63-OH
eRF1 K63-H
relative abundance
elution time (min)
C
% O2
21 1 0.1 0
HIF-1α
Jmjd4
eRF1
β-actin
21% O2
1% O2
0.1% O2
0% O2
>95% hydroxylation
~90% hydroxylation
~50% hydroxylation
<2% hydroxylation
eRF1 K63-OH
eRF1 K63-H
relative abundance
elution time (min)

Figure S3, related to Figure 3. eRF1 K63 hydroxylation is dependent on 2OG and oxygen. (**A**) eRF1 was immunopurified from HEK293T cells prior to Arg-C proteolysis. MS/MS spectrum of an Arg-C peptide (m/z 977.508) containing residues 48-65 confirms hydroxylation at K63, as shown by peptide fragments from both y- (blue) and b- (red) ion series. (**B**) Dimethyloxalylglycine (DMOG) inhibits eRF1 K63 hydroxylation. HeLa cells were either untreated or treated with 1mM DMOG, a cell permeable 2OG analogue inhibitor, for 24 hours and simultaneously labelled with a heavy isotope of L-lysine ($^{13}C_6$; Lys^6) (SILAC methodology) to allow LC-MS quantitation of K63 hydroxylation in newly-synthesized eRF1. Left panel: immunoblot of extracts from treated cells confirming successful DMOG treatment, as indicated by HIF-1α induction following HIF prolyl hydroxylase inhibition (Epstein et al., 2001). Right panel: LC-MS analyses of trypsin-digested eRF1 quantitating K63 hydroxylation. Extracted ion chromatograms are shown for the SILAC labelled trypsin peptide containing residues 51-63 ($[M+H]^{2+}$; K63-H: m/z 701.852; K63-OH: m/z 709.850). (C) eRF1 K63 hydroxylation is oxygen-dependent. HeLa cells were cultured in normoxia (21% oxygen), hypoxia (1% or 0.1% oxygen) or anoxia (0% oxygen) for 24h and simultaneously labelled with a heavy isotope of L-lysine ($^{13}C_6$; Lys^6) to allow LC-MS quantitation of K63 hydroxylation in newly-synthesized eRF1. Left: immunoblot of extracts from treated cells confirming successful hypoxia or anoxia treatment, as indicated by HIF-1α induction following HIF prolyl hydroxylase inhibition (Epstein et al., 2001). Right panel: LC-MS analyses of trypsin-digested eRF1 quantitating K63 hydroxylation. Extracted ion chromatograms are shown for the trypsin peptide containing residues 51-63 ($[M+H]^{2+}$; K63-H: m/z 701.852; K63-OH: m/z 709.850).

Figure S4

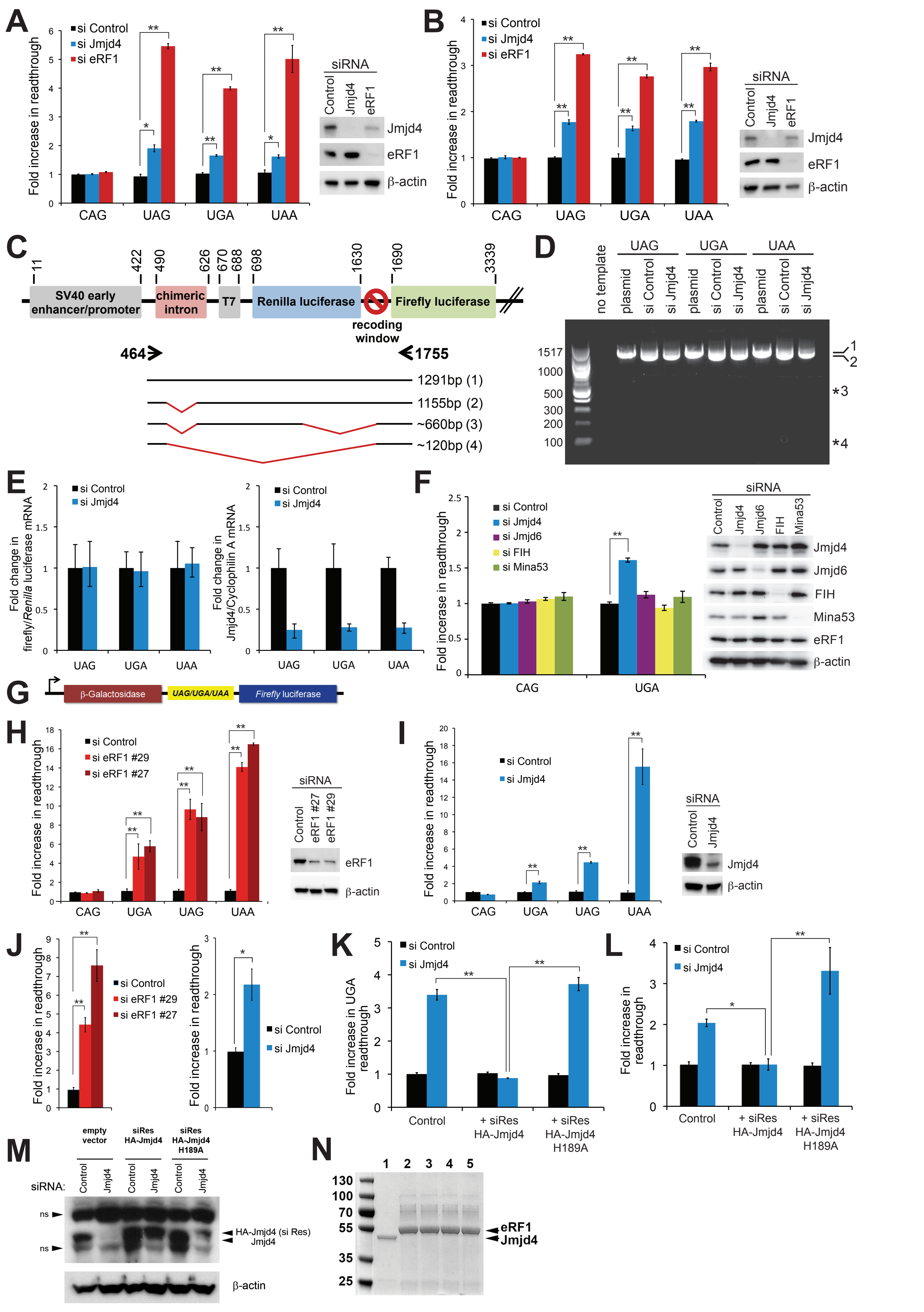
A
si Control
si Jmjd4
si eRF1
Fold increase in readthrough
CAG
UAG
UGA
UAA
siRNA
Control
Jmjd4
eRF1
β-actin
B
C
SV40 early enhancer/promoter
chimeric intron
T7
Renilla luciferase
Firefly luciferase
recoding window
464
1755
1291bp (1)
1155bp (2)
~660bp (3)
~120bp (4)
D
no template
plasmid
si Control
si Jmjd4
E
Fold change in firefly/Renilla luciferase mRNA
Fold change in Jmjd4/Cyclophilin A mRNA
F
si Jmjd6
si FIH
si Mina53
Jmjd6
FIH
Mina53
Fold incease in readthrough
G
β-Galactosidase
UAG/UGA/UAA
Firefly luciferase
H
si eRF1 #29
si eRF1 #27
eRF1 #27
eRF1 #29
I
J
K
Fold increase in UGA readthrough
Control
+ siRes HA-Jmjd4
+ siRes HA-Jmjd4 H189A
L
M
empty vector
siRes HA-Jmjd4
siRes HA-Jmjd4 H189A
siRNA:
ns
HA-Jmjd4 (si Res)
N
eRF1
Jmjd4

Figure S4, related to Figure 4. Jmjd4 knockdown induces stop codon readthrough in multiple cell types and contexts. (**A**) Jmjd4 knockdown in U2OS cells induces readthrough of a *Renilla*:stop:firefly reporter in which the stop codon is embedded within a leaky termination sequence from tobacco mosaic virus. Left Panel: U2OS cells were transfected with the reporters in Figure 4B prior to siRNA (si) transfection and dual luciferase assay. Right Panel: Immunoblot of reporter extracts indicating Jmjd4 and eRF1 knockdown. (**B**) Similar analyses in (A) performed in Hep3B cells. (**C**) Schematic of the *Renilla*-Firefly luciferase bicistronic vector used in this study (Grentzmann et al., 1998), indicating normal splicing to remove the chimeric intron from the full-length mRNA (1) to produce product (2). Sequences inserted within the recoding window have potential to generate cryptic splice sites and aberrant splice variants of product (2) (see [Holcik et al., 2005; Lemp et al., 2012]) indicated as products (3) and (4). Primers (indicated by arrow heads) were designed to span from the chimeric intron to firefly luciferase in order to detect the indicated transcripts and potential splice products. (**D**) RT-PCR analyses demonstrate the integrity of the *Renilla*:stop:firefly mRNA. Total RNA was purified from HeLa cells transfected with control or Jmjd4 siRNA and the bicistronic luciferase reporters depicted in (C), prior to DNase digest to remove plasmid DNA followed by cDNA synthesis and end point RT-PCR. Purified plasmid DNA was used as a positive control ('plasmid'). Note the absence of lower molecular weight splice variants indicating that the integrity of the reporter has not been compromised by insertion of the TMV termination sequence. (**E**) Left Panel: qPCR demonstrating that the abundance of firefly relative to *Renilla* luciferase mRNA is not altered by Jmjd4 siRNA. Right Panel: siRNA knockdown of Jmjd4 was confirmed by qPCR in parallel to analysis of the bicistronic reporter RNA (**F**) Closely-related JmjC hydroxylases do not regulate translational termination. Left Panel: HeLa cells were transfected with the *Renilla*:UGA:firefly

reporter prior to the indicated siRNA (si) transfection and dual luciferase assay. Right Panel: Immunoblot of reporter extracts indicating knockdown of the indicated JmjC hydroxylases. (**G**) Schematic of an alternative stop codon readthrough reporter: β-galactosidase:stop:firefly luciferase. (**H**) Transfection of A549 cells with two independent eRF1 siRNA sequences induces stop codon readthrough. Left Panel: A549 cells were transfected with the reporters in (G) prior to siRNA (si) transfection followed by β-galactosidase and firefly luciferase assays. Right Panel: Immunoblot of reporter extracts indicating eRF1 knockdown. (**I**) Transfection of A549 cells with Jmjd4 siRNA induces stop codon readthrough. Left Panel: A549 cells were transfected with the reporters in (G) prior to siRNA (si) transfection followed by β-galactosidase and firefly luciferase assays. Right Panel: Immunoblot of reporter extracts indicating Jmjd4 knockdown. Note that a single siRNA sequence is presented since the specificity is confirmed in (K) and (L). (**J**) Transfection of A549 cells with eRF1 (left) or Jmjd4 (right) siRNA also induces readthrough of the β-galactosidase:stop:firefly luciferase reporter containing a stop codon embedded within a strong termination sequence. See (H) and (I) for corresponding immunoblots. (**K**) Jmjd4 hydroxylase activity is required for efficient translational termination. Stop codon readthrough assays were performed as in (H) using A549 cell lines stably expressing empty vector, siRes-HA-Jmjd4, or siRes-Jmjd4 H189A mRNAs, and the β-galactosidase:stop:firefly luciferase reporter with an UGA stop codon embedded within tobacco mosaic virus termination sequence. (**L**) Jmjd4 hydroxylase activity is also required for efficient translational termination at a strong termination sequence. Stop codon readthrough assays were performed as in (H) using A549 cell lines stably expressing empty vector, siRes-HA-Jmjd4, or siRes-Jmjd4 H189A mRNAs, and the β-galactosidase:stop:firefly luciferase reporter with a strong termination sequence. (**M**) Immunoblot analyses of extracts from cells treated in (K) and (L) confirming knockdown of

endogenous Jmjd4 and re-expression of siRNA-resistant Jmjd4 (n.s. = non-specific immunoreactivity). (**N**) Coomassie-stained gel of recombinant proteins demonstrate that Jmjd4-reacted eRF1 samples were successfully depleted of contaminating Jmjd4 before measuring their release factor activity in Figures 4E and 4F. The lanes are as follows: (1) Recombinant wildtype Jmjd4 (to highlight the position of potential contaminant in lanes 2 to 5). The amount of Jmjd4 loaded is equivalent to the relative amount in the bulk hydroxylation reaction prior to eRF1 purification. (2) Wildtype eRF1 purified after bulk hydroxylation reaction with wildtype Jmjd4. (3) Wildtype eRF1 purified after bulk hydroxylation reaction with Jmjd4-H189A. (4) eRF1 K63R purified after bulk hydroxylation reaction with wildtype Jmjd4. (5) eRF1 K63R purified after bulk hydroxylation reaction with Jmjd4-H189A. In panels showing bicistronic reporter results, data are presented as mean ± S.E.M. Statistical significance was determined by paired two-tail Student's t-test (I, J) for single comparisons, and analysis of variance (ANOVA) followed by Dunett's (A, B, F, H, J) or Bonferroni (K, L) post-hoc tests for multiple comparisons ($*P<0.05$; $**P<0.01$).

Supplemental Experimental Procedures

Cell culture and treatments

Stable HEK293T cells overexpressing FLAG-Jmjd4 or FLAG-FIH were cultured as described in the main text, in the presence of 1μg/ml puromycin. HeLa cell lines stably expressing pTIPZ doxycycline-inducible siRNA-resistant HA-Jmjd4 (wildtype or H189A mutant; see below) were cultured under the same conditions as parental HeLa cells, except that Tet system-approved fetal calf serum (Clontech) was used in addition to 1μg/ml puromycin. RNA interference (Mission siRNA, Sigma) was performed using two to three consecutive transfections with Oligofectamine (for A549 and HeLa; Invitrogen) or N-TER (for U2OS; Sigma) reagent at 25nM siRNA per transfection. Transient transfections were performed using Fugene6 (HeLa and U2OS; Roche) or Turbofect (A549; Fermentas) according to the manufacturer's instructions. Endogenous eRF1 hydroxylation was measured in SILAC-labelled HeLa cells (see below) treated as follows. Hypoxia was performed at 1, 0.1 or 0% oxygen for 24 hours using a Ruskinn hypoxia station, followed by cell lysis (see below) in the presence of 1mM N-oxalylglycine (NOG; Santa Cruz Biotechnology). Cellular inhibition of 2OG oxygenases was achieved by treating cells with 1mM dimethyl-N-oxalylglycine (DMOG; Frontier Scientific) for 24 hours.

Expression constructs and reporters

Site-directed mutagenesis was performed by PCR using standard procedures. Human Jmjd4 and FIH cDNAs were cloned into the Not-I/Cla-I restriction sites of the pGIPZ vector (Open Biosystems) using PCR primers incorporating N-terminal 3XFLAG epitope tags, thereby replacing the GFP cDNA with the required enzyme cDNA. The human Jmjd4 cDNA was cloned

into the pTriEx2 recombinant expression vector platform (Merck) by PCR. β-galactosidase:*stop*:firefly luciferase reporters were designed to encode the β-galactosidase cDNA upstream of a recoding window and in-frame with the firefly luciferase cDNA. Plasmids were constructed using a two-step PCR cloning strategy: the β-galactosidase coding sequence was inserted into pEF1/V5-HisA (Invitrogen) via KpnI and BamHI sites, and the firefly luciferase gene subsequently inserted *via* BamHI and NotI restriction sites with a 5' extension on the forward primer containing the recoding sequences. To induce basal readthrough stop codons were positioned in the context of a minimal leaky termination signal derived from the replicase cistron of the plant tobacco mosaic virus (TMV) (Pelham, 1978): CAA [STOP: UAG/UGA/UAA] CAA UUA. Controls included either replacing the UAG stop codon with a sense CAG codon for continuous readthrough, or substituting the leaky termination signal for a strong stop codon: UGU [UAA] AGG AAA. All constructs were sequence verified prior to use.

Statistical analyses

Data were analyzed using IBM SPSS Statistics software version 21.0 and MATLAB (Mathworks) version 7.13.0.564. Paired two-tailed Student's t-test was performed for single comparisons. For multiple comparisons, analysis of variance (ANOVA) was performed followed by Dunnett's or Bonferroni post-hoc analysis. All results are presented as mean ± SEM. To assess whether the bicistronic reporter data were normally distributed, the probability plot correlation coefficient (PPCC) test for normality was used (Filliben, 1975; Jacobs and Dinman, 2004). An example probability plot is shown below which visualizes how closely the data matches a standard normal distribution (data corresponds to Figure S4B). The PPCC quantifies

the level of this correlation. All calculated PPCCs were >0.869 indicating that there is no significant deviation from normality.

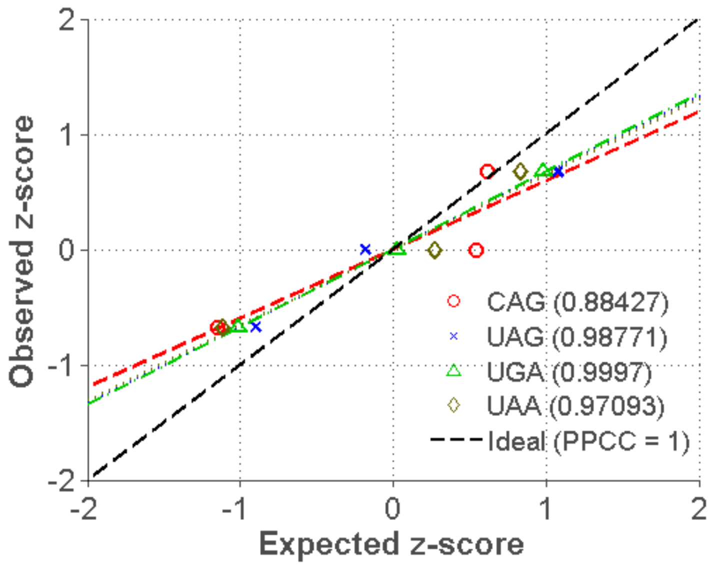

Immunoblotting

Following SDS-PAGE samples were electroblotted to polyvinylidene fluoride membrane (Millipore). Membranes were blocked in 5% (w/v) milk powder in PBS/0.1% Tween-20 prior to probing with the following primary antibodies; anti-HA-HRP (Roche), anti-FLAG-HRP (Sigma), anti-Jmjd4 (Sigma), anti-eRF1 (Santa Cruz Biotechnology), anti-FIH (Stolze et al., 2004), anti-Jmjd6 (Santa Cruz Biotechnology), anti-MINA53 (Invitrogen) and anti-β-actin-HRP (Abcam). HRP-conjugated secondary antibodies were purchased from Dako. Signals were developed using SuperSignal West Pico, Dura, or Femto chemiluminescent substrates (Thermo Fisher Scientific).

Mapping and quantitation of hydroxylation sites by proteomics

Jmjd4 pulldowns to identify candidate substrates were performed by immunoprecipitating extracts derived from control, FLAG-Jmjd4, or FLAG-Jmjd4 H189A overexpressing cells with anti-FLAG affinity resin (Sigma) overnight at 4°C. Identification of hydroxylation sites in the eRF1/eRF3a complex was performed by first transiently transfecting HEK293T cells with V5-eRF1 and HA-eRF3a in the presence or absence of HA-Jmjd4 for 48 hours, prior to immunoprecipitation of extracts with anti-V5 affinity resin (Sigma) overnight at 4°C. Immunocomplexes from both of the above experiments were thoroughly washed in lysis buffer prior to elution in ammonium hydroxide and precipitation with methanol/chloroform. Endogenous eRF1 was purified from various sources by extracting samples in lysis buffer prior to immunoprecipitation with mouse anti-eRF1 monoclonal antibody (Santa Cruz Biotechnology) overnight at 4°C. These immunocomplexes were thoroughly washed in lysis buffer prior to elution in Laemmli buffer, SDS-PAGE separation, and coomassie blue staining. All samples were then digested in bicarbonate buffer using sequencing-grade trypsin (Sigma) or Arg-C (Roche) (Cockman et al., 2009). SILAC was performed by pulse-labelling cells with lysine-deficient DMEM (Thermo Fisher Scientific) containing 10% (v/v) dialyzed fetal bovine serum (Appleton Woods) supplemented with 'heavy' L-lysine (U-$^{13}C_6$; Lys^6; Cambridge Isotope Laboratories) for the duration of the treatment.

Mass spectrometry analysis

Samples were resuspended in 20 µl of Buffer A (2% acetonitrile, 0.1% formic acid) and subjected to LC-MS/MS (Waters, Acquity, 75 µm x 250 mm, 1.7 µm particle size) analysis using a Thermo LTQ Orbitrap Velos (Thermo Scientific) at a Resolution of 30000. MS/MS

spectra were acquired in CID mode, selecting up to 20 precursors. Peptides were separated by a linear gradient of 1-40% Acetonitrile in 120 min at a flow rate of 250nl/min (Fischer et al., 2012). MS/MS spectra were extracted from raw files by ProteoWizard MSConvert software (http://proteowizard.sourceforge.net/) using the 200 most intense peaks in each and converted into MGF files. The peaklists were searched against the IPI human database (v3.87, 91464 entries) using Mascot (http://www.matrixscience.com/) v2.3.01, allowing one missed cleavage and 20ppm/0.5 Da mass deviations in MS MS/MS. Carbamidomethylation of cysteine was a fixed modification. Oxidation of methionine and lysine as well as deamidation of asparagine and glutamine were variable modifications. Annotation of oxidized methionines and lysines was performed manually and assisted with ModLS (Trudgian et al., 2012) as part of data analysis using the Central Proteomics Facilities Pipeline (Trudgian et al., 2010).

RNA analyses

Total RNA was extracted from HeLa cells using TRIzol (Sigma) following the manufacturer's instructions. To remove contaminating plasmid DNA, extracted RNA was treated with DNAse turbo (Ambion) prior to cDNA synthesis by reverse transcriptase (Applied Biosystems). End point RT-PCR assays to monitor integrity of the Renilla-stop-firefly mRNA were performed using Q5 high-fidelity DNA polymerase (New England Biolabs) and the following primers: forward 5'-CAGAAGTTGGTCGTGAGGCA-3' and reverse 5'-TCCAGCGGTTCCATCCTCTA -3'. PCR products were separated by electrophoresis on 1.5% w/v agarose/TBE gels and visualised using ethidium bromide and UV. To measure the relative abundance of *Renilla* and firefly luciferase mRNAs, qRT-PCR were performed using SYBR green dye (Applied Biosystems), with the following primers for *Renilla* luciferase: forward 5'-

AAGAGCGAAGAGGGCGAGAA -3' and reverse 5'-TGCGGACAATCTGGACGAC-3', for firefly luciferase: forward 5'-CGTGCCAGAGTCTTTCGACA-3' and reverse 5'-ACAGGCGGTGCGATGAG-3'. Jmjd4 mRNA abundance was quantified by qRT-PCR using a TaqMan probe (Applied Biosystems) and analysed using the StepOnePlus Real Time PCR System (Applied Biosystems) corrected against Taqman control Cyclophilin A.

***In vitro* hydroxylation assays with recombinant eRF1 and Jmjd4**

Small scale reactions were undertaken as follows. Recombinant eRF1 (2ug) and Jmjd4 (200ng) were incubated at 37°C in 200µl of assay buffer (50mM Tris-HCl pH 7.4, 16µM Fe[II], 160µM 2OG, and 1mM DTT) in the presence or absence of 1mM NOG. After the indicated incubation time, the reaction was quenched with 0.1% formic acid. Proteins were extracted using methanol-chloroform, digested with trypsin and eRF1 K63 hydroxylation quantitated by LC-MS.

Batch scale reactions for *in vitro* translation assays were performed as follows. Bacterial pellets expressing recombinant protein were lysed in 50mM Tris-HCl pH8, 200mM KCl, 0.5% Triton-X-100, 10% glycerol, 0.2mg/ml lysozyme, protease inhibitors and 6mM β-mercaptoethanol, before sonication and centrifugation to remove insoluble debris. Supernatants were mixed in a 1:10 volume ratio of Jmjd4 to eRF1 prior to four separate additions of 10mM 2OG and 80µM Fe(II) over a 16 hour period at room temperature. eRF1 was subsequently purified by a two-step chromatography method using Ni-NTA and MonoQ columns as described in the main text.

Components of the fully reconstituted *in vitro* translation system

The 40S and 60S ribosomal subunits, as well as eukaryotic translation factors eIF2, eIF3, eEF1 and eEF2, were purified from a rabbit reticulocyte lysate as described (Alkalaeva et al., 2006). The eukaryotic translation factors eIF1, eIF1A, eIF4A, eIF4B, eIF4G, eIF5B, eIF5, eRF1 and its K63R mutant were produced as recombinant proteins in *E. coli* strain BL21 with subsequent protein purification on Ni-NTA agarose and ion-exchange chromatography (Alkalaeva et al., 2006). MVHL-stop transcription vectors have been described (Alkalaeva et al., 2006).

Pre-termination complex assembly and purification

Pre-termination complexes (pre-TC) were assembled as described (Alkalaeva et al., 2006). Briefly, 37 pmol of MVHL-stop mRNAs were incubated for 30 min in buffer A (20 mM Tris acetate, pH 7.5, 100 mM KOAc, 2.5 mM $MgCl_2$, 2 mM DTT) supplemented with 400 U RNase inhibitor (RiboLock, Fermentas), 1 mM ATP, 0.25 mM spermidine, 0.2 mM GTP, 75 μg total tRNA (acylated with Val, His, Leu and [^{35}S]Met), 75 pmol 40S and 60S purified ribosomal subunits, 125 pmol eIF2, eIF3, eIF4G, eIF4A, eIF4B, eIF1, eIF1A, eIF5, eIF5B each, 200 pmol eEF1 and 50 pmol eEF2 and then centrifuged in a Beckman SW55 rotor for 95 min at 4°C and 50 000 rpm in a 10%–30% linear sucrose density gradient prepared in buffer A with 5 mM $MgCl_2$. Fractions corresponding to pre-TC complexes according to optical density and the presence of [^{35}S]Met were combined, diluted 3-fold with buffer A containing 1.25 mM $MgCl_2$ (to a final concentration of 2.5 mM Mg^{2+}) and used in termination efficiency assays.

Termination efficiency assay

Termination efficiency (k_{cat}/K_M) was determined from k_{obs} as described (Eliseev et al., 2011). Briefly, aliquots containing 0.0125 pmol of pre-TC assembled in the presence of [^{35}S]Met-tRNA were incubated at 37°C with 2.5 pmol of eRF1 for 0-15 min. Ribosomes and tRNA were pelleted with ice-cold 5% TCA supplemented with 0.75% casamino acids and centrifuged at 4°C and 14 000 g. The amount of released [^{35}S]Met-containing tetrapeptide was used for determination of the efficiency of peptidyl-tRNA hydrolysis.

Peptide synthesis

The eRF1 24mer linear peptide (LADEFGTASNIKSRVNRLSVLGAI) was commercially synthesized by G.L. Biochem (Shanghai, China). For synthesis of the cyclic eRF1 peptide, a linear precursor peptide (Gly-Thr-Ala-Ser-Asn-Ile-Lys-Ser-Arg-Val-Asn-Arg-Leu-Ser-[D-Cys]) was prepared by standard solid phase synthesis on a CS Bio CS336X peptide synthesizer (100 μmol scale) using DIC as coupling reagent. After cleavage of the *N*-terminal Fmoc-protecting group, a solution of 150 mg of chloromethylcarbonyloxysuccinimide (ClAc-OSu) in 4 mL DMF was added to the resin and the mixture was shaken for 3 h. The resin was filtered off and subsequently treated with 4 mL of deprotection solution (95 % TFA, 2.5 % Triisopropylsilane, 2.5 % water). After 3 h the volume was reduced to 1 mL under a nitrogen stream and the peptides were crashed out with cold Et_2O. The mixture was centrifuged and the supernatant discarded. The solid was taken up in 1.5 mL of triethylammoniumacetate buffer (1 M, pH 8.5) and the pH readjusted to >8. The mixture was heated in a microwave (Biotage Initiator) to 80°C for 10 min, and the cyclic peptide was subsequently purified by HPLC (0-45 % MeCN in 45 min, 0.1 % TFA, Dionex Ultimate 3000 series, Grace Vydac 218TP101522 column):

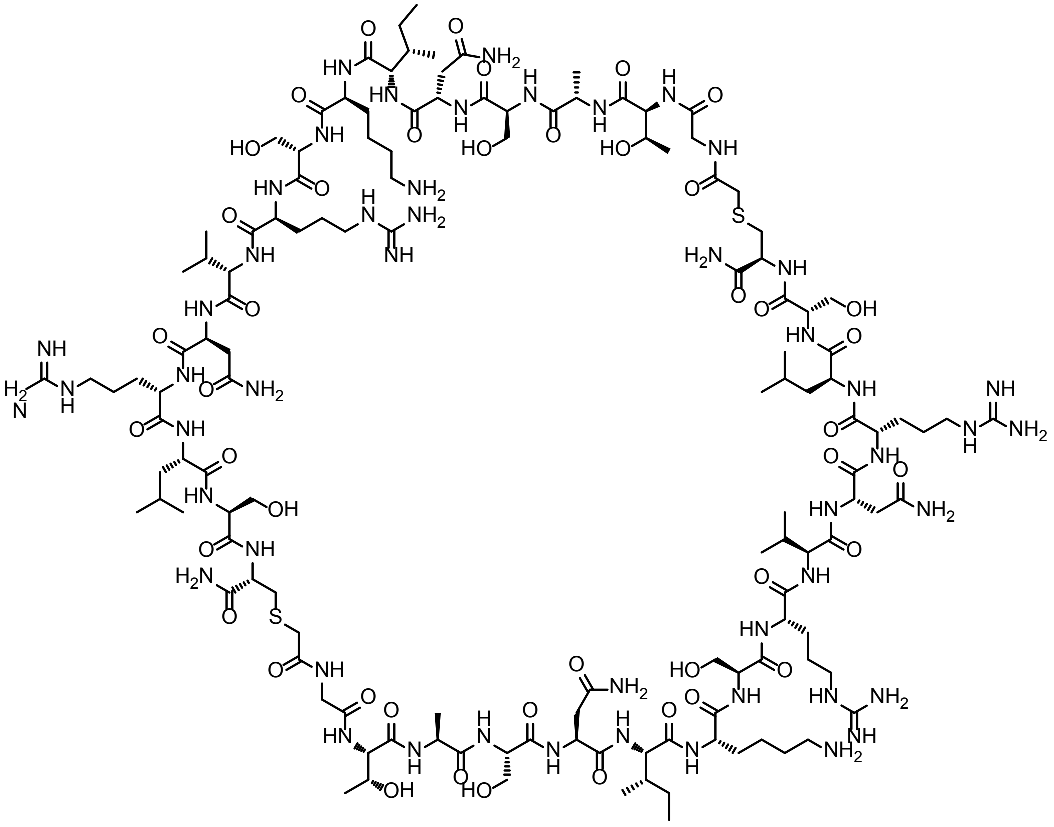

HRMS (ESI+): calculated for $C_{132}H_{236}N_{50}O_{44}S_2^{2+}$ $[M+2H^+]^{2+}$: 1644.8599, found: 1644.8481

Sample preparation for MALDI analysis

eRF1 peptides (50 μM) were incubated at 37 °C with recombinant Jmjd4 (10 μM), Fe(II) (100 μM) and 2OG (200 μM) in 50 mM HEPES pH 7.5, or with Jmjd6 as described (Webby et al., 2009). Reactions were quenched after 1 hour by the addition of 0.1 % formic acid, and 1 μL of the resultant mixture was spotted directly onto a MALDI target plate with 1 μL α-cyano-4-hydroxycinnamic acid matrix. MALDI analyses were carried out using a MALDI-TOF micro MX™ mass spectrometer (Waters Micromass™). Mass spectra were acquired in positive ion reflectron mode with a 17 kV acceleration voltage.

Sample preparation for amino acid analysis

For amino acid analyses, the eRF1 cyclic peptide (50 μM) was incubated with recombinant Jmjd4 (10 μM), Fe(II) (100 μM) and 2OG (200 μM) for 1 hour at 37 °C in HEPES buffer (50 mM, pH 7.5). The reaction was quenched by the addition of methanol (50 % final concentration), incubated on ice for 2 hours and then centrifuged to pellet any precipitated Jmjd4. The supernatant containing the cyclic peptide (as confirmed by MALDI-TOF MS analysis) was transferred to a fresh tube and dried in a vacuum centrifuge. The dried reaction mixture was then resuspended in double distilled water and the peptide subjected to enzymatic hydrolysis at 37 °C with trypsin (1 μL, 100 μg mL^{-1}; Promega) for 1 h, followed by Carboxypeptidase Y (1 μL, 5 mg mL^{-1}, Roche) overnight.

The hydrolyzed peptides were dried by vacuum centrifugation, reconstituted in borate buffer pH 9.0 and derivatized by 6-aminoquinolyl-*N*-hydroxysuccinimidyl carbamate (AQC) in acetonitrile according to the AccQ-Tag Ultra Derivatization Protocol (Waters). LC-MS analyses were performed as described (Mantri et al., 2011) using a Waters Acquity ultra performance liquid chromatography system coupled to an LCT Premier XE orthogonal acceleration time-of-flight mass spectrometer equipped with an electrospray ionization source (Waters, USA). The gradient conditions used for separation were as follows:

t [min]	*flow rate*	% Solvent A (AccQ Tag Ultra Phase A)	% Solvent B (98.7% acetonitrile, 1.3% formic acid)
0.00	0.7	99.9	0.1
25.00	0.6	90.0	10.0
25.50	0.6	40.0	60.0
27.50	0.6	40.0	60.0
28.00	0.6	100.0	0.0

30.00	0.7	100.0	0.0

The conditions for ESI-MS detection were as follows: positive-ion mode, V-mode analyser, desolvation temperature at 250 °C, source temperature at 120 °C, capillary voltage at 3000 V, sample cone voltage at 100 V, cone and desolvation gas flow at 30 and 550 L/min, respectively. The MS data were acquired and an extracted ion chromatogram produced for the m/z 503.1, corresponding to bis-derivatised hydroxylysine. The trace was smoothed (Savitzky-Golley, 2 iterations, 3 points window).

Synthesis of 3-hydroxylysine

3-Hydroxylysine was synthesized as summarized in Scheme 1. Initially, 4-aminobutanal diethylacetal was protected as the phtalimide. The aldehyde was subsequently liberated using mild acid hydrolysis. In the key step ethylisocyanoacetate was reacted with the aldehyde using transition metal catalysis. The resulting dihydrooxazole was cleaved with HCl; we did not acquire evidence for elimination within limits of detection. In the final step the phtalimide was cleaved using hydrazine to give 3-hydroxylysine as a mixture of stereoisomers:

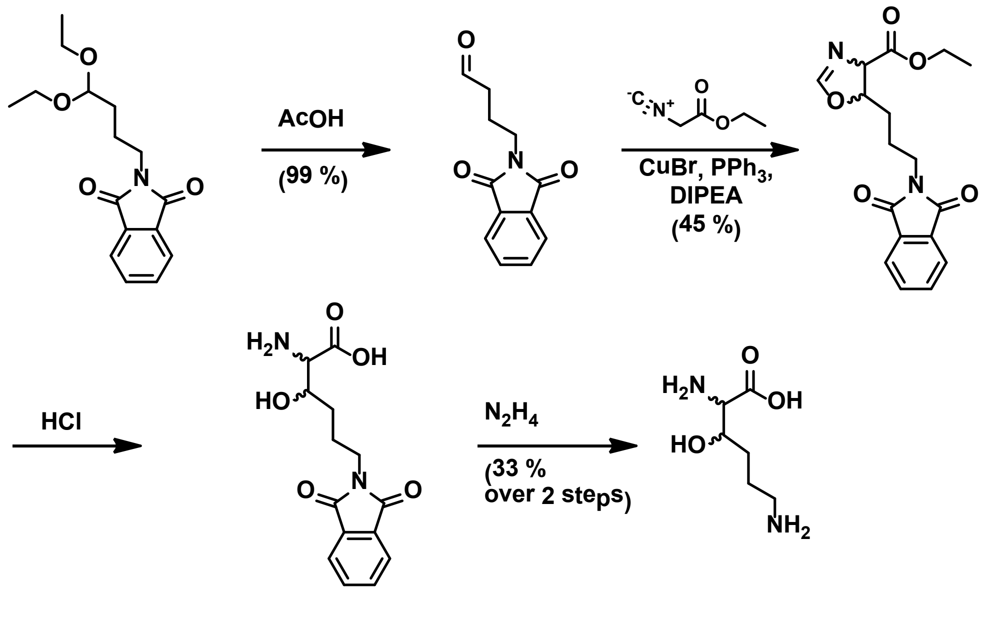

Scheme 1: Synthesis of 3-hydroxylysine.

2-(4,4-Diethoxybutyl)isoindoline-1,3-dione

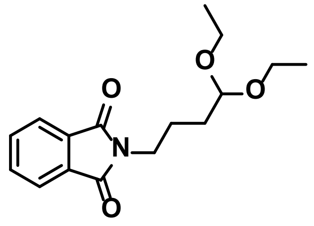

N-Ethoxycarbonylphtalimide (8.10 g, 36.6 mmol, 1.05 mmol) was dissolved in 40 mL CH_2Cl_2, 4-aminobutanal dimethylacetal (6.00 mL, 35.0 mmol, 1.0 eq) and NEt_3 (5.40 mL, 36.6 mmol, 1.05 eq) were added and the reaction mixture stirred at room temperature for 4 h. After completion of the reaction the solvent was evaporated *in vacuo* to give 7.92 g (78 %) of 2-(4,4-diethoxybutyl)isoindoline-1,3-dione (Teshima et al., 1991).

^{1}H NMR ($CDCl_3$, 400MHz): δ = 7.82 (dd, *J*=5.5, 3.0 Hz, 1 H), 7.69 (dd, *J*=5.5, 3.0 Hz, 1 H), 4.70 (broad singlet, 1 H), 4.50 (t, *J*=5.5 Hz, 1 H), 4.10 (q, *J*=7.0 Hz, 1 H), 3.70 (t, *J*=7.0 Hz, 1

H), 3.62 (dq, J=9.5, 7.1 Hz, 2 H), 3.47 (dq, J=9.5, 7.1 Hz, 2 H), 3.14 (q, J=7.5 Hz, 1 H), 1.70 - 1.80 (m, 1 H), 1.60 - 1.69 (m, 2 H), 1.33 (t, J=7.5 Hz, 1 H), 1.24 (t, J=7.0 Hz, 2 H), 1.17 ppm (t, J=7.0 Hz, 4 H)

^{13}C NMR ($CDCl_3$,101MHz): δ = 168.4, 133.8, 132.1, 123.1, 102.4, 61.2, 37.7, 30.9, 23.9, 15.3 ppm

HRMS (ESI+): calculated for $C_{16}H_{21}NNaO_4^+$ $[M+H^+]^+$: 314.1363, found:314.1353.

4-(1,3-Dioxoisoindolin-2-yl)butanal

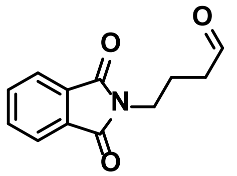

2-(4,4-Diethoxybutyl)isoindoline-1,3-dione (500 mg, 1.72 mmol, 1.0 eq) was dissolved in 10 mL H_2O and CH_2CO_2H (20.0 mL, 343 mmol, 200 eq) was added. The reaction mixture was stirred for 2.5 h at room temperature, then concentrated *in vacuo* to afford 427 mg (yield apparently quantitative, but residual CH_2CO_2H present) of 4-(1,3-dioxoisoindolin-2-yl)butanal (Iradier et al., 2001).

^{1}H NMR ($CDCl_3$, 400MHz): δ = 9.76 (s, 1 H), 7.79 - 7.86 (m, 2 H), 7.67 - 7.74 (m, 2 H), 3.73 (t, J=7.0 Hz, 2 H), 2.53 (t, J=7.5 Hz, 2 H), 2.00 ppm (quin, J=7.0 Hz, 2 H)

^{13}C NMR ($CDCl_3$, 101MHz): δ = 200.8, 168.3, 134.0, 131.9, 123.2, 41.0, 37.1, 21.1 ppm

HRMS (ESI+): calculated for $C_{12}H_{11}NNaO_3^+$ $[M+Na^+]^+$: 240.0631, found: 240.0629

Ethyl 5-(3-(1,3-dioxoisoindolin-2-yl)propyl)-4,5-dihydrooxazole-4-carboxylate

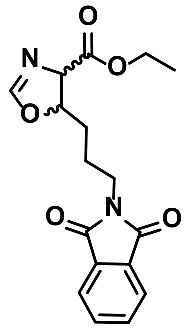

4-(1,3-Dioxoisoindolin-2-yl)butanal (100 mg, 0.46 mmol, 1.0 eq), CuBr (7 mg, 0.05 mmol, 0.1 eq) and PPh_3 were (24 mg, 0.09 mmol, 0.1 eq) were dissolved in 5 mL degassed CH_2Cl_2. Diisopropylethylamine (DIPEA) (8 µL, 0.05 mmol, 0.1 eq) and ethylisocyanoacetate (53 µL, 0.46 mmol, 1.0 eq) were added and the reaction mixture stirred at room temperature for 2 h. Subsequently the solvent was removed *in vacuo* and the crude product purified by column chromatography (0 – 15 % MeOH in DCM, flat gradient) to give 46 mg (45 %) of ethyl 5-(3-(1,3-dioxoisoindolin-2-yl)propyl)-4,5-dihydrooxazole-4-carboxylate as a mixture of diastereomers. As indicated by MS-analysis, the C-2 formamide resulting from dihydrooxazole hydrolysis was observed as a substantial byproduct (Benito-Garagorri et al., 2006).

^{1}H NMR ($CDCl_3$, 400MHz): δ = 8.28 (s, 1 H), 7.81 (dd, *J*=5.5, 3.0 Hz, 3 H), 7.69 (dd, *J*=5.5, 3.0 Hz, 3 H), 6.73 (d, *J*=9.0 Hz, 1 H), 4.68 (d, *J*=8.5 Hz, 1 H), 4.20 (qt, *J*=7.0, 3.5 Hz, 4 H), 3.70 (t, *J*=7.0 Hz, 3 H), 3.25 (d, *J*=5.0 Hz, 1 H), 2.07 (broad singlet, 1 H), 1.70 - 1.94 (m, 3 H), 1.50 - 1.58 (m, 3 H), 1.26 ppm (t, *J*=7.0 Hz, 6 H).

^{13}C NMR ($CDCl_3$, 101MHz): δ = 170.5, 169.7, 168.5, 164.5, 161.5, 134.0, 131.9, 128.6, 128.5, 123.2, 123.2, 77.3, 76.7, 72.3, 71.4, 62.0, 61.9, 60.4, 56.5, 55.0, 37.4, 30.7, 30.1, 25.1, 24.9, 21.0, 14.1, 14.0 ppm.

HRMS (ESI+): calculated for $C_{17}H_{19}N_2O_5^+$ $[M+H^+]^+$: 331.1288, found: 331.1294

2-Amino-6-(1,3-dioxoisoindolin-2-yl)-3-hydroxyhexanoic acid

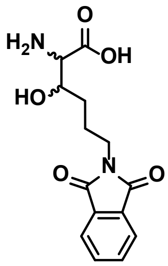

Ethyl-5-(3-(1,3-dioxoisoindolin-2-yl)propyl)-4,5-dihydrooxazole-4-carboxylate (46 mg, 0.15 mmol, 1.0 eq) was dissolved in 10 mL HCl (6 M) and heated to reflux overnight, then cooled and concentrated *in vacuo*. The crude product was used directly in the next reaction.

^{1}H NMR (D_2O, 400MHz): δ = 7.61 - 7.69 (m, 4 H), 7.53 (dd, *J*=5.5, 3.5 Hz, 2 H), 4.07 - 4.16 (m, 1 H), 3.86 - 3.93 (m, 1 H), 3.55 (t, *J*=6.5 Hz, 1 H), 2.89 - 2.97 (m, 1 H), 2.59 (s, 1 H), 1.68 - 1.81 (m, 1 H), 1.57 - 1.68 (m, 2 H), 1.45 - 1.57 ppm (m, 2 H).

^{13}C NMR (D_2O, 101MHz): δ = 172.1, 171.1, 135.0, 133.5, 132.3, 131.6, 129.1, 125.0, 123.6, 68.8, 39.4, 37.5, 30.6, 30.3, 24.4, 23.7 ppm

2,6-Diamino-3-hydroxyhexanoic acid (3-hydroxylysine)

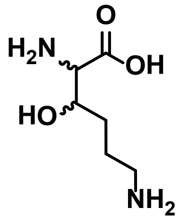

2-Amino-6-(1,3-dioxoisoindolin-2-yl)-3-hydroxyhexanoic acid (40 mg, 0.13 mmol, 1.0 eq) was dissolved in 5 mL H_2O, $N_2H_4.H_2O$ (27 mg, 0.33 mmol, 2.5 eq) was then added. The reaction mixture was stirred at room temperature for 18 h, then purified by HPLC (0-100 % MeCN in

H_20, 0.1 %CF_3CO_2H) to give 8 mg (33 % over 2 steps) of 3-hydroxylysine as a mixture of diastereomers (see Figure S2D).

^{1}H NMR (D_2O, 400MHz): δ = 4.01 - 4.07 (m, 1 H), 3.78 (d, *J*=4.5 Hz, 1 H), 2.88 - 2.95 (m, 2 H), 1.69 - 1.79 (m, 1 H), 1.57 - 1.69 (m, 2 H), 1.45 - 1.57 ppm (m, 1 H).

HRMS (ESI+): calculated for $C_6H_{15}N_2O_3^+$ $[M+H^+]^+$: 163.1077, found: 163.1077.

Synthesis of 4-Hydroxylysine

4-Hydroxylysine was synthesized as a mixture of all 4 stereoisomers using a modified literature procedure (Morin et al., 1998). 4,5-Dehydrolysine dihydrochloride (Bachem, 10 mg, 0.05 mmol) was dissolved in 6M H_2SO_4 (5 mL) and the solution was heated under reflux for 18 hours. The pH of the solution was then adjusted to pH 10 using 5M NaOH and used directly for amino acid analysis. A ^{1}H NMR spectrum of the reaction mixture revealed the presence of 4-hydroxylysine (see Figure S2E).

^{1}H NMR (700 MHz, D_2O) 1.59-1.74 (m, 3H, H_β, H_δ), 1.77-1.83 (m, 1H, H_β), 2.76-2.85 (m, 2H, H_ε), 3.43 (app q, *J* = 4.5 Hz, 1H, H_α), 3.81-3.89 (m, 1H, H_γ).

^{13}C NMR (175 MHz, D_2O) 36.9 (C_δ, C_ε), 41.2 (C_β), 53.0 (C_α), 66.5 (C_γ).

Supplemental References

Alkalaeva, E.Z., Pisarev, A.V., Frolova, L.Y., Kisselev, L.L., and Pestova, T.V. (2006). In vitro reconstitution of eukaryotic translation reveals cooperativity between release factors eRF1 and eRF3. Cell *125*, 1125-1136.

Benito-Garagorri, D., Bocokic, V., and Kirchner, K. (2006). Copper(I)-catalyzed diastereoselective formation of oxazolines and N-sulfonyl-2-imidazolines. Tetrahedron Letters *47*, 8641-8644.

Cockman, M.E., Webb, J.D., Kramer, H.B., Kessler, B.M., and Ratcliffe, P.J. (2009). Proteomics-based identification of novel factor inhibiting hypoxia-inducible factor (FIH) substrates indicates widespread asparaginyl hydroxylation of ankyrin repeat domain-containing proteins. Molecular & cellular proteomics : MCP *8*, 535-546.

Eliseev, B., Kryuchkova, P., Alkalaeva, E., and Frolova, L. (2011). A single amino acid change of translation termination factor eRF1 switches between bipotent and omnipotent stop-codon specificity. Nucleic Acids Res *39*, 599-608.

Epstein, A.C., Gleadle, J.M., McNeill, L.A., Hewitson, K.S., O'Rourke, J., Mole, D.R., Mukherji, M., Metzen, E., Wilson, M.I., Dhanda, A., *et al.* (2001). C. elegans EGL-9 and mammalian homologs define a family of dioxygenases that regulate HIF by prolyl hydroxylation. Cell *107*, 43-54.

Filliben, J.J. (1975). The Probability Plot Correlation Coefficient Test for Normality. Technometrics *17*, 111-117.

Fischer, R., Trudgian, D.C., Wright, C., Thomas, G., Bradbury, L.A., Brown, M.A., Bowness, P., and Kessler, B.M. (2012). Discovery of candidate serum proteomic and metabolomic biomarkers in ankylosing spondylitis. Molecular & cellular proteomics : MCP *11*, M111 013904.

Grentzmann, G., Ingram, J.A., Kelly, P.J., Gesteland, R.F., and Atkins, J.F. (1998). A dual-luciferase reporter system for studying recoding signals. RNA *4*, 479-486.

Holcik, M., Graber, T., Lewis, S.M., Lefebvre, C.A., Lacasse, E., and Baird, S. (2005). Spurious splicing within the XIAP 5' UTR occurs in the Rluc/Fluc but not the betagal/CAT bicistronic reporter system. RNA *11*, 1605-1609.

Iradier, F., Arrayas, R.G., and Carretero, J.C. (2001). Synthesis of medium-sized cyclic amines by selective ring cleavage of sulfonylated bicyclic amines. Organic letters *3*, 2957-2960.

Jacobs, J.L., and Dinman, J.D. (2004). Systematic analysis of bicistronic reporter assay data. Nucleic Acids Res *32*, e160.

Lemp, N.A., Hiraoka, K., Kasahara, N., and Logg, C.R. (2012). Cryptic transcripts from a ubiquitous plasmid origin of replication confound tests for cis-regulatory function. Nucleic Acids Res *40*, 7280-7290.

Mantri, M., Loik, N.D., Hamed, R.B., Claridge, T.D., McCullagh, J.S., and Schofield, C.J. (2011). The 2-oxoglutarate-dependent oxygenase JMJD6 catalyses oxidation of lysine residues to give 5S-hydroxylysine residues. Chembiochem : a European journal of chemical biology *12*, 531-534.

Morin, B., Bubb, W.A., Davies, M.J., Dean, R.T., and Fu, S. (1998). 3-Hydroxylysine, a potential marker for studying radical-induced protein oxidation. Chemical research in toxicology *11*, 1265-1273.

Pelham, H.R. (1978). Leaky UAG termination codon in tobacco mosaic virus RNA. Nature *272*, 469-471.

Stolze, I.P., Tian, Y.M., Appelhoff, R.J., Turley, H., Wykoff, C.C., Gleadle, J.M., and Ratcliffe, P.J. (2004). Genetic analysis of the role of the asparaginyl hydroxylase factor inhibiting hypoxia-

inducible factor (FIH) in regulating hypoxia-inducible factor (HIF) transcriptional target genes [corrected]. J Biol Chem *279*, 42719-42725.

Teshima, T., Matsumoto, T., Wakamiya, T., Shiba, T., Aramaki, Y., Nakajima, T., and Kawai, N. (1991). Total Synthesis of nstx-3, spider toxin of nephila maculata. Tetrahedron *47*, 3305-3312.

Trudgian, D.C., Singleton, R.S., Cockman, M.E., Ratcliffe, P.J., and Kessler, B.M. (2012). ModLS: Post-translational modification localization scoring with automatic specificity expansion. J. Proteomics Bioinform *5*, 283-289.

Trudgian, D.C., Thomas, B., McGowan, S.J., Kessler, B.M., Salek, M., and Acuto, O. (2010). CPFP: a central proteomics facilities pipeline. Bioinformatics *26*, 1131-1132.

Webby, C.J., Wolf, A., Gromak, N., Dreger, M., Kramer, H., Kessler, B., Nielsen, M.L., Schmitz, C., Butler, D.S., Yates, J.R., 3rd*, et al.* (2009). Jmjd6 catalyses lysyl-hydroxylation of U2AF65, a protein associated with RNA splicing. Science *325*, 90-93.
